# Intrinsically disordered regions of nucleophosmin/B23 regulate its RNA binding activity through their inter- and intra-molecular association

**DOI:** 10.1093/nar/gkt897

**Published:** 2013-10-06

**Authors:** Miharu Hisaoka, Kyosuke Nagata, Mitsuru Okuwaki

**Affiliations:** Faculty of Medicine, University of Tsukuba, 1–1–1 Tennoudai, Tsukuba 305–8575, Japan

## Abstract

Nucleophosmin (NPM1/B23) is a nucleolar protein implicated in growth-associated functions, in which the RNA binding activity of B23 plays essential roles in ribosome biogenesis. The C-terminal globular domain (CTD) of B23 has been believed to be the RNA binding domain because the splicing variant B23.2 lacking the CTD binds considerably less efficiently to RNA. However, the recognition of target RNAs by B23 remains poorly understood. Herein, we report a novel mechanism by which B23 recognizes specific RNA targets. We observed that the nucleolar retention of B23.3 lacking the basic region of B23.1 was lower than that of B23.1 because of its low RNA binding activity. Circular dichroism measurements indicated that the basic region and adjacent acidic regions of B23 are intrinsically disordered regions (IDRs). Biochemical analyses revealed that the basic IDR alone strongly binds to RNA with low specificity. The excessive RNA binding activity of the basic IDR was restrained by intra-molecular interaction with the acidic IDR of B23. Chemical cross-linking experiments and fluorescent labeling of bipartite tetracysteine-tagged proteins suggested that the inter- and intra-molecular interactions between the two IDRs contribute to the regulation of the RNA binding activity of CTD to control the cellular localization and functions of B23.

## INTRODUCTION

The biological activities of proteins are often exerted by structurally ordered domains; therefore, structure-function analyses have been central in understanding several biological processes. However, recent studies have documented that the structurally disordered flexible domain in the native state [intrinsically disordered regions (IDRs)] play crucial roles in the regulation of protein–protein and protein–nucleic acid interactions (1). The IDRs often adopt a structured conformation on binding to their target molecules. For example, the N-terminal transactivation domain in the transcription factor p53 is intrinsically disordered and folded on binding to the Taz2 domain of the co-activator, p300 (2). In addition, several DNA binding proteins containing zinc fingers, helix–loop–helix motifs and homeodomains have N- or C-terminal extended IDRs that assist efficient recognition and binding to target sequences (3). Furthermore, various post-translational modifications occur in the IDRs, suggesting that the IDRs play an integral role in regulation of the protein functions (4).

The nucleolar phosphoprotein NPM1/B23 is involved in the regulation of pre-ribosome RNA (rRNA) transcription, processing and pre-ribosome transport (5–7). In addition, B23 plays important roles in the regulation of centrosome duplication (8), genomic stability (9) and response to cellular stresses (10). Because of its wide range of functions, the deregulation of B23 functions is closely associated with tumorigenesis (10). Mutations of the C-terminal domain (CTD) of B23 are frequently observed in normal karyotype adult acute myeloid leukemia (11). Moreover, B23 overexpression is often observed in solid tumors (12–17). Therefore, it is crucial to understand the mechanism of B23 action.

**Figure 1.**
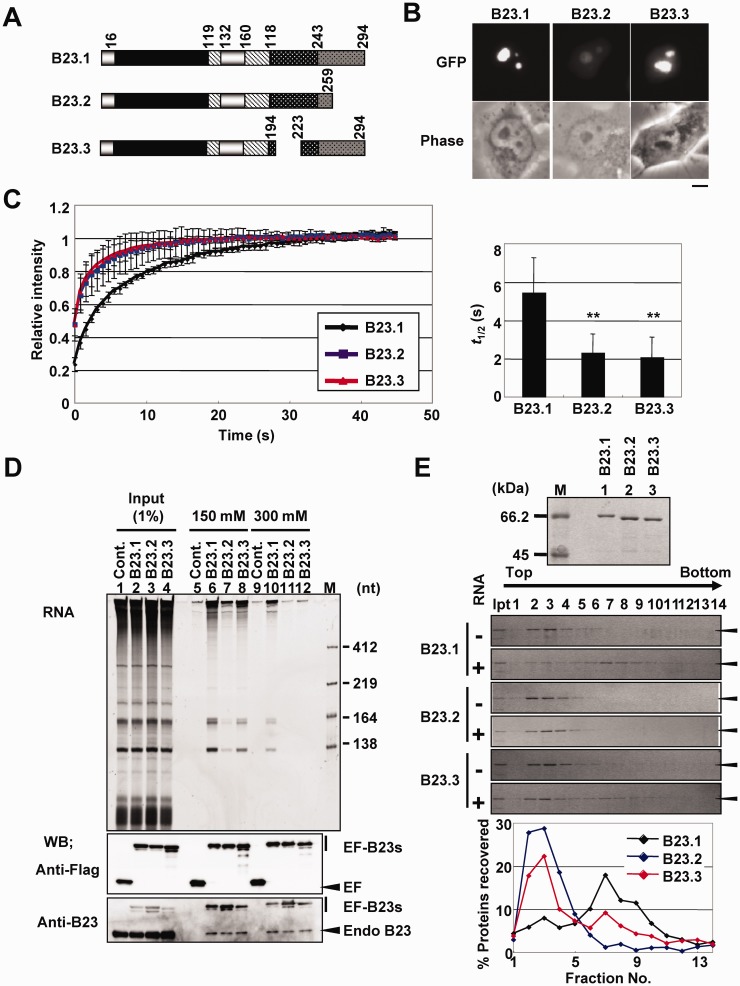
Characterization of B23 variants. (**A**) Schematic representation of B23 splicing variants. Black, oligomerization domain; stripes, acidic regions; black with white dots, basic region; dark gray with black dots, CTD. (**B**) Localization of EF-tagged B23 variants. HeLa cells were transfected with vectors for expression of EF-B23.1, EF-B23.2 and EF-B23.3. Localization of the protein was observed by fluorescent microscope. Bar at the bottom indicates 10 µm. (**C**) FRAP analysis of B23 variants. The mobility of EF-B23 proteins expressed in HeLa cells as in Figure 1B were examined by FRAP assay. The fluorescence at the bleached nucleoli relative to that before bleaching (1.0) was calculated and plotted as a function of time. The data were represented as mean values ± SD from 15, 15 and 13 experiments for B23.1 (black), B23.2 (blue) and B23.3 (red), respectively. The *t*_1/2_ of fluorescence recovery was estimated by curve fitting and graphically represented (right panel). *P*-values were calculated by *t*-tests and indicated with **P < 0.01. (**D**) Immunoprecipitation of EF-tagged B23 variants. 293T cells expressing EF, EF-B23.1, EF-B23.2 and EF-B23.3 (lanes 1–4, respectively) were subjected to immunoprecipitation with anti-Flag tag antibody in the buffer containing 150 mM (lanes 5–8) or 300 mM (lanes 9–12) of NaCl. Precipitated proteins were separated by SDS–PAGE and detected with western blotting using anti-Flag tag, and -B23 antibodies (bottom panels). RNAs co-precipitated with EF-tagged proteins were purified and separated on 6% denaturing PAGE and visualized by Gel Red staining (top panel). Lane M indicates RNA molecular markers. (**E**) RNA binding activity of B23 variants. Recombinant GST-tagged B23 proteins were analyzed by SDS–PAGE followed by CBB staining (top panel). Purified proteins (5 µg) were incubated in the absence or presence of total RNA (10 µg) prepared from 293T cells and loaded on a 15–40% sucrose gradient. Proteins in fractions collected from the top were analyzed by SDS–PAGE and visualized by CBB staining. For the experiments incubated with RNA, the amount of the B23 proteins in each fraction relative to that of input was calculated and shown in graph (bottom panel).

We previously identified B23 as a factor involved in adenovirus chromatin remodeling (18). B23 was found to be involved not only in adenovirus chromatin remodeling but also in the regulation of the cellular *rRNA* gene chromatin to stimulate transcription (5). B23 mainly localizes in the nucleolus but shuttles between the nucleolus, nucleoplasm and cytoplasm. Three splicing variants of B23 were reported to be expressed in human cells (19) (see Figure 1A). B23.1 is the longest variant and the most studied protein among the B23 variants; B23.2 has 259 amino acids and lacks the C-terminal 35 amino acids (dark gray bar with black dots); the third variant, which we have named B23.3, has 265 amino acids and lacks 29 amino acids in the basic amino acid-rich region (shown as black bar with white dots in Figure 1A). B23 forms a pentamer and decamer through the N-terminal oligomerization domain (black bar in Figure 1A) (20). A recent study reported that the CTD of B23.1 forms globular structure consisting of three α-helices, and its destabilization abolishes its nucleolar localization (21). Because B23.2 does not bind to RNA, CTD is believed to be crucial for its RNA binding activity (22). It was also shown that the CTD of B23 binds to G-quadruplex DNA, and this activity is reinforced by at least 17 adjacent residues located in the basic region (23,24). Several prediction programs suggest that the acidic and basic regions in the central part of B23 are IDRs. The two acidic regions [acidic IDR (aIDR), stripe bar in Figure 1A] are important for the histone chaperone activity of B23; however, the structure of this region and relationship to its RNA binding activity have not been analyzed. Recently, it was reported that a synthetic peptide derived from the basic region of B23, which shows a typical random-coil spectrum by circular dichroism (CD), stabilizes its CTD (25). We previously demonstrated that the cdc2/cyclin B kinase phosphorylates the basic IDR (bIDR) of B23.1, and this phosphorylation significantly decreases its RNA binding activity (26). However, the mechanism by which cdc2/cyclin B-mediated phosphorylation decreases the RNA binding activity of B23.1 is currently unknown.

In this study, we examined the bIDR involved in the regulation of the RNA binding activity of B23.1. We observed that B23.3 lacking a part of the bIDR showed significantly lower RNA binding activity than B23.1. Further analyses strongly suggested that the association between acidic and bIDRs of B23.1 allowed its recognition and binding to target RNAs.

## MATERIALS AND METHODS

### Cell culture and transfection

HeLa and 293T cells were maintained in Dulbecco’s modified Eagle’s medium (Nacalai Tesque) supplemented with 10% fetal bovine serum. Transient transfection of plasmid DNA was performed using GeneJuice (Novagen) according to the manufacturer’s instructions.

Antibodies Anti-B23 serum was generated in rabbit using the His-B23.1-CR protein as an antigen. Anti-Flag tag antibody (M2, SIGMA-ALDRICH) and anti-His tag antibody (HIS-1, SIGMA-ALDRICH) were commercially available.

Plasmids pEGFPC1-Flag-B23.1, pGEX2T-B23.1, and pET14b-B23.1, pET14b-B23.1-ΔC1 (used as B23.2), pET14b-B23.1-ΔC2 (used as B23.1-C188), pET14b-B23.1-ΔC3, and pEGFPC1-Flag-B23.2, pET14b-B23.2, and pGEX2T-B23.2 and pET14b-B23.1-T4sD were described previously (18,26–28). To construct pEGFP-Flag-B23.3, the cDNA fragment was generated using a two-step PCR based on the sequence NM_199185. In the first PCR, two DNA fragments for B23.1 (1–194) and B23.1 (233–294) were amplified using T7 promoter primer and 5′-AGTGAAGAAAGGACAAGAATCCTTCAAG-3′ and T3 promoter primer and 5′-ATTCTTGTCCTTTCTTCACTGGCGCTTT-3′, with pBS-Flag-B23.1 as a template. The amplified DNA fragments were used in the second PCR as templates with T7 and T3 promoter primers. Amplified DNA fragment was digested by NdeI and BamHI and cloned into the same sites of pET14b. The B23.3 cDNA was cloned into pBS-Flag by NdeI and HindIII sites of pBS-Flag vector. The Flag-B23.3 cDNA was cut out from pBS-Flag-B23.3 by digestion with BamHI and subcloned into the same sites of pGEX2T (GE Healthcare) or pEGFPC1 (Clonetech Laboratories). To construct the expression vectors of GST- or His-tag fusion protein if not otherwise specify, cDNAs prepared as following were digested by BamHI or NdeI and BamHI, and cloned into the same sites of pGEX2T or pET14b, respectively. To construct pGEX2T-B23.1-CR, pGEX2T-B23.1-CRA, pGEX2T-B23.1-CRD and pGEX2T-B23.1-CR1, the cDNA fragments were amplified by PCR using 5′-AAGGATCCCATATGAAAGCGCCAGTGAAGAAA-3′ and T7 terminator primer, with appropriate templates. The B23.1-CR1.5 cDNA was amplified using 5′-AAGGATCCCATATGAGTTCTGTAGAAGACATTAA-3′ and GEX-3′ primer, and pGEX2T-B23.1-CR as a template. To construct pGEX2T-B23.1-ΔA2, the cDNA fragment was generated using a two-step PCR. Two DNA fragments were amplified using the T7 promoter primer and 5′-CTGGCGCTTTAGCAGCAAGTTTTACTTT-3′ and the T3 promoter primer and 5′-ACTTGCTGCTAAAGCGCCAGTGAAGAAA-3′, with pBS-Flag-B23.1 as a template. The second PCR and cloning into pGEX2T of this fragment was performed as described earlier in the text. To construct pGEX2T-ΔA1A2, the cDNA was cut out from pBS-Flag-B23-ΔA (5) by digestion with BamHI and subcloned into the same site of pGEX2T. The B23.1-ΔN and B23.2-ΔN cDNA fragments were amplified using 5′-AAGAATTCAACATATGGAGGAAGATGCAGAGTCAGA-3′ and T7 terminator primer, and pET14b-B23.1 and pET14b-B23.2 as templates. To construct pET14b-B23.1-Nc, N-terminal Cys–Cys- (CC-) tag was introduced by PCR using 5′-AAGGATCCATATGTTGAACTGCTGCGAGGAAGATGCAGAGTCA-3′ and T7 terminator primer, and pET14b-B23.1-ΔN as a template. To construct pET14b-B23.1-Cc and pET14b-B23.1-NCcc, Cys–Cys- (CC-) tag was introduced by PCR using 5′-GCAGCAAGGTCCTTTTGGTGTTTTAGGAG-3′ and 5′-GTAGAAGACATTAAAGCAAAAATG-3′, and pET14b-B23.1-ΔN and pET14b-B23.1-Nc as templates. To construct pET14b-B23.1-Ccc, tetracysteine (CCPGCC) tag was introduced by PCR using 5′-GCAGCATCCTGGGCAGCAGTTCAAAGGTCCTTTTGGTGTTTTAGGAGT-3′ and 5′-GTAGAAGACATTAAAGCAAAAATG-3′, and pET14b-B23.1-ΔN as a template. Amplified DNA fragments containing pET14b vector sequence were phosphorylated with T4 polynucleotide kinase (TOYOBO) for following ligation reaction. pET14b-B23.2-Nc, pET14b-B23.2-Cc, pET14b-B23.2-NCcc and pET14b-B23.2-Ccc were constructed with the same procedure as pET14b-B23.1-ΔN, using T7 promoter primer and 5′-GCCTAAGGATCCTTAACCACCTTTTTCTATACTTGC-3′, and appropriate vectors as templates. The fragments of pET14b-B23.1-C160, -C194, -C206, -C223 and -C241, and pET14b-B23.1-ΔA-C160, -C194, -C206, -C223 and -C241 were amplified using T7 promoter primer and 5′-ccggatccttaagcagcaagttttacttttttctg-3′, 5′-ccggatccttatttcttcactggcgctttttc-3′, 5′-ccggatccttacttttgtgcatttttggctgg-3′, 5′-ccggatccttattttgatcttggtgttgatga-3′ and 5′-ccggatccttaaggtccttttggtgttttagg-3′, and pET14b-B23.1 and pET14b-B23.1-ΔA1A2 as templates, respectively.

### Purification of recombinant proteins

For expression and purification of recombinant proteins, BL21 (DE3) was transformed with pGEX2T or pET14b plasmids containing B23.1 mutant cDNAs. The transformed *Escherichia coli* were grown at 37°C until OD_600_ reached 0.4. Expression of the recombinant proteins was induced by addition of isopropyl β-D-thiogalactopyranoside at 30°C for 3 h or at 18°C for 16 h. For purification of GST-tagged proteins, sonicated bacterial cell lysates were incubated with Glutathione sepharose (GE Healthcare) in buffer A [50 mM Tris–HCl (pH 7.9), 0.1% Triton X-100 and 0.1 mM PMSF] containing 150 mM NaCl. The resin was washed with buffer A containing 500 mM NaCl, treated with micrococcal nuclease (MNase) (Nuclease S7, Roche Applied Science), and extensively washed with buffer A containing 500 mM NaCl. GST-tagged proteins retained on the resin were eluted in a buffer containing [20 mM reduced glutathione, 100 mM Tris–HCl (pH 7.9) and 120 mM NaCl]. His-tagged proteins were purified with HIS-Select® Nickel affinity gel according to the method suggested by the manufacturer (SIGMA-ALDRICH). Purified proteins were dialyzed against buffer H [20 mM Hepes–NaOH (pH 7.9), 50 mM NaCl, 0.5 mM EDTA, 1 mM DTT, 0.1 mM PMSF and 10% Glycerol]. His-tagged proteins used in proteinase digestion assays and FlAsH assays were further purified by separation on SDS–PAGE followed by extraction in a buffer containing [0.1% SDS, 50 mM Tris–HCl (pH 7.9), 0.1 mM EDTA, 150 mM NaCl and 5 mM DTT] and precipitated with acetone. Precipitated proteins were denatured in a buffer containing [6 M guanidine–HCl, 50 mM Tris–HCl (pH 7.9) and 5 mM DTT], refolded by dialysis against a buffer containing [50 mM Hepes–NaOH (pH 8.0), 12.5 mM MgCl_2_, 100 mM KCl, 0.2 mM EDTA, 0.1% NP40 and 1 mM DTT] and dialyzed against buffer H. The protein concentrations were estimated by comparison with proteins in a standard molecular mass marker (Bio-Rad) on SDS–PAGE stained with Coomassie Brilliant Blue (CBB). Identity of a part of recombinant proteins was confirmed by matrix-assisted laser-desorption time-of-flight mass spectrometry (MALDI-TOF-MS), and the purity of the proteins was also analyzed by SDS–PAGE (Supplementary Figure S1). The phosphorylation of recombinant proteins was performed as described in (26), using mitotic cell extracts as an enzyme source in a buffer containing [20 mM Tris–HCl (pH 7.4), 10 mM MgCl_2_, 50 mM NaCl, 3 mM ATP, 1 mM β-D-glycerophosphate, 1 mM NaVO_4_ and 1 mM NaF] at 37°C for 1 h. After phosphorylation reaction, CaCl_2_ (final 5 mM) and MNase (Nuclease S7, Roche Applied Science) were added, and proteins were incubated for additional 30 min followed by purification using GST-tag as described earlier in the text. Phosphorylation status was examined by phospho affinity gel electrophoresis using phos-tag® (Wako Pure Chemical Industries) (29) in the presence of Mn^2+^.

### Immunofluorescence and fluorescence recovery after photobleaching analysis

To observe the localization of B23 splicing variants, HeLa cells were transiently transfected with EF-tagged B23 vectors. Eighteen hours after transfection, cells were analyzed by fluorescence microscope (Olympus, IX71N-22FL/PH-TS). For fluorescence recovery after photobleaching (FRAP) analysis, HeLa cells were transiently transfected with EF-B23.1, EF-B23.2 and EF-B23.3, and grown on 35-mm glass base dishes (IWAKI). The dish was set on inverted microscope (LSM EXCITER; Carl Zeiss Microimaging, Inc.) in an air chamber containing 5% CO_2_ at 37°C and analyzed as previously described (30). The data were represented as mean values ± SD from 15, 15, and 13 experiments for B23.1, B23.2, and B23.3, respectively.

### Immunoprecipitation

293T cells were transiently transfected with EF, EF-B23.1, EF-B23.2 and EF-B23.3 with Gene Juice (Novagene). Sixteen hours after transfection, the cells were collected and sonicated in buffer A containing 150 mM NaCl. The cell lysates were incubated with anti-Flag M2 affinity gels (SIGMA-ALDRICH) in buffer A containing 150 mM or 300 mM NaCl and 35 units RNase inhibitor (Nacalai Tesque). The resins were washed with the same buffer, and treated with 10 units of DNase I (Invitrogen), and washed extensively with the buffer same. The proteins bound with the resin were eluted with buffer A containing 150 mM NaCl and Flag peptide (SIGMA-ALDRICH), separated by SDS–PAGE and analyzed by western blotting. RNAs co-precipitated with EF-tagged proteins were treated with proteinase K, purified with phenol-chloroform extraction and ethanol precipitation and separated by 6% denatured PAGE. RNAs were visualized by Gel Red staining (Biotium).

### Sucrose density gradient centrifugation assays

GST-tagged B23 proteins (5 µg) preincubated in the absence or presence of total RNA purified from 293 T cells (10 µg) or total RNA alone (10 µg) (100 µl) were loaded on 15–45% sucrose density gradient in the buffer containing [20 mM Hepes–NaOH (pH 7.9), 50 mM NaCl and 0.5 mM EDTA] (2.1 ml). The samples were centrifuged at 54 000 rpm for 2 h at 4°C in S55S rotor (Hitachi Koki, SC100GXII), and fractions (150 µl) were collected from the top. Proteins in each fraction were separated by SDS–PAGE and visualized by CBB staining. RNAs in each fraction were purified, separated by 1% denatured agarose gel and visualized by Gel Red staining. The distribution patterns of proteins and RNAs were analyzed using CS analyzer (ATTO).

### CD spectral analysis

CD spectral analyses were performed using a JASCO spectrometer J-710 instrument (JASCO Inc.) at room temperature in the wavelength interval 190–260 nm. Data were collected at 0.2 nm resolution, 20 nm/min scan speed, 1.0 nm bandwidth and 2 s response using 0.1 cm path-length quartz cuvette. Data for each protein collected from four scans were averaged. The signal was converted to mean molar residue ellipticity in units of mdeg cm^2^ dmol^−^^1^. The concentration of His-tagged B23 proteins was 5 µM in 50 mM sodium phosphate buffer (pH 7.0).

### Filter binding assays

Filter binding assays were performed as described (26). RNAs used in this assay was purified from 293 T cells using Sepasol® RNA I Super G (Nacalai tesque) and treated with DNase I. Experiments performed with doublet were repeated more than twice. The data were represented as mean values ± SD.

### Mobility shift assays

GST-B23.1-A was incubated with GST-tagged B23.1-CR1, B23.1-CR1.5 and B23.1-CR in 10 µl of buffer H for 30 min at room temperature. Incubated samples were separated on 6% native-PAGE in 0.5× TBE and visualized by CBB staining.

### Trypsin digestion assays

His-B23.1 and His-B23.1 -ΔA1A2 (2 µg) were incubated in 10 µl of 50 mM phosphate buffer (pH 7.6) containing Trypsin for 2 min at 37°C. The proteolysis was stopped with the addition of 3.5 µl of 4×SDS sample buffer and incubation for 5 min at 95°C, and proteins were separated by SDS–PAGE followed by western blotting using anti-His tag antibody.

### FlAsH labeling

Recombinant proteins containing Cys-tag (1.1, 2.2, 4.4, 6.6, 8.8 and 13.2 µM, respectively) were mixed with 10 µM FlAsH-EDT_2_ (Toronto Research Chemicals) and 0.5 µM EDT (Nacalai Tesque) in a buffer containing [10 mM Hepes–NaOH (pH 8.0), 100 mM NaCl, 1 mM EDTA, 25 mM Tris–HCl (pH 7.9)] in 384-well plate (Corning). The fluorescence emission at 535 ± 5 nm was detected by Varioskan (Thermo Fisher Scientific).

### Cross-link assays

His-B23.1 -ΔN and His-B23.2 -ΔN (15–45 pmoles) were incubated in the buffer [20 mM Hepes–NaOH (pH 7.9), 50 mM NaCl, 0.5 mM EDTA, 10% Glycerol, 1mM DTT, 0.5 mM PMSF] containing 0.1% glutaraldehyde (10 µl) for 10 min at room temperature. The reaction was stopped by the addition of 1 µl of 1M Tris–HCl (pH 7.9) and 3.5 µl of 4 × SDS–PAGE sample buffer, and incubation at 95°C for 5 min. Cross-linked proteins were separated by SDS–PAGE and visualized by CBB staining.

## RESULTS

### Localization and RNA binding activity of B23 splicing variants

Three splicing variants of NPM1/B23, B23.1, B23.2 and B23.3 are expressed in HeLa cells (Figure 1A). The third variant, which we named B23.3, has not been characterized thus far. We first characterized the cellular localization and biochemical activity of this variant protein compared it with that of B23.1 and B23.2. EGFP-Flag (EF)-tagged B23.1, B23.2 and B23.3 were transiently expressed in HeLa cells, and their localization was observed (Figure 1B). Consistent with previous observations (26), EF-B23.1 was primarily detected in the nucleolus, whereas EF-B23.2 was detected in both the nucleoplasm and nucleolus. EF-B23.3 was primarily localized in the nucleolus, and it was also clearly detected in the nucleoplasm. We subsequently examined the nucleolar retention of B23 splicing variants using FRAP assays (Figure 1C). EGFP signal in a small nucleolus was targeted for bleaching with a 488-nm laser line, and the intensity of EGFP was measured every 0.5 s. The recovery curves indicated that the mobility of B23.2 was considerably faster than that of B23.1, and the half time (*t*_1/2_) of EF-B23.1 and -B23.2 recovery was 5.48 ± 1.80 s and 2.33 ± 0.96 s, respectively. This finding was consistent with the previous observation that CTD plays a crucial role in the nucleolar retention of B23 (21,26). Despite intact CTD, the mobility of B23.3 was similar to that of B23.2 (*t*_1/2_ = 2.11 ± 1.02 s).

Localization and chromatin association of B23 are closely related to its RNA binding activity (26,28). Therefore, we examined the RNA binding activity of B23 splicing variants in cells (Figure 1D). EF-tagged B23 proteins were transiently expressed in 293T cells, and immunoprecipitation assays were performed. Under the assay conditions, EF-tagged B23 proteins were equally precipitated (Figure 1D, bottom panels). RNAs co-precipitated with EF-tagged proteins were extracted and analyzed by denaturing polyacrylamide gel and agarose gel electrophoresis (Supplementary Figure S2A). EF-B23.1 co-precipitated with 28S, 5.8S and 5S rRNAs as previously reported (28), but not with 18S rRNA, whereas B23.2 did so inefficiently (lanes 6 and 7). rRNA association of EF-B23.3 was significantly lower than that of EF-B23.1 in the presence of 150 mM NaCl and was hardly detectable in the presence of 300 mM NaCl (lanes 10 and 12). The 28S, 5.8S and 5S rRNA binding activity of EF-B23.3 was approximately half compared with that of EF-B23.1 in the presence of 150 mM NaCl (Supplementary Figure S2B). To investigate the direct RNA binding activity, we performed sucrose density gradient centrifugation assays using GST-tagged recombinant proteins and total RNAs purified from 293T cells (Figure 1E). All three proteins were recovered from the low-density fractions in the absence of RNA (Figure 1E, lanes 2–4). In the presence of RNA, GST-B23.1 was primarily recovered from the 28S rRNA peak fractions (Figure 1E, lanes 6–10 and Figure 3C). In contrast, fractions of GST-B23.2 did not clearly change, regardless of the presence of RNA. Although a minor population of GST-B23.3 was recovered from the 28S rRNA peak fractions, the fractionation pattern of GST-B23.3 was not significantly changed by the presence of RNA. Because the fractionation pattern of GST alone was not affected by the presence of RNA and non-tagged and GST-tagged B23.1 showed similar fractionation pattern in the presence of RNA (Supplementary Figure S3A and B), GST-tagging did not affect the RNA binding activity of B23 proteins. These results indicate that not only CTD but also the region lacking in B23.3 play important roles in the RNA binding activity of full-length B23.1 in cells and *in vitro*.

### Central regions of B23 are intrinsically disordered

**Figure 2.**
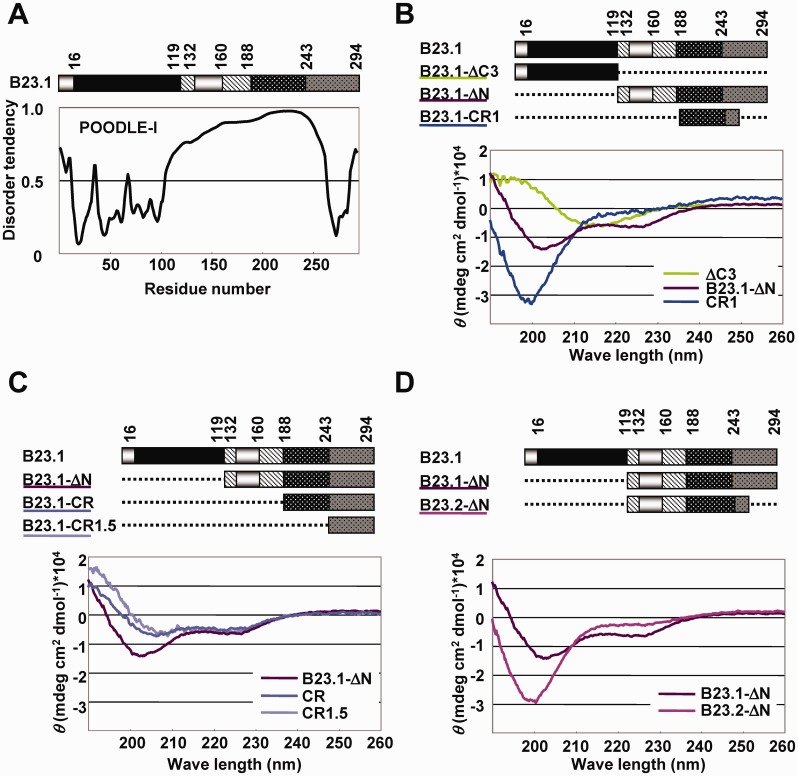
Structural analysis of aIDR and bIDR. (**A**) Prediction of disorder tendency of B23.1. B23.1 structure was schematically represented at the top. The patterns in the scheme are as defined in the legend of Figure 1A. Disorder tendency of B23.1 was predicted using POODLE-I. (**B–D**) CD spectral analyses of B23 mutants. His-tagged B23 mutant proteins (5 µM) were analyzed. His-B23.1-ΔC (green), His-B23.1-ΔN (purple) and His-B23.1-CR1 (blue) (B). His-B23.1-ΔN (purple), His-B23.1-CR (blue) and His-B23.1-CR1.5 (light purple) (C). His-B23.1-ΔN (purple) and His-B23.2-ΔN (pink) (D).

Recent studies showed that the IDRs regulate the functions of structured regions, such as protein–protein or protein–nucleic acid binding activity (1). The acidic and basic regions of B23 have low mean hydrophobicity and high net charge, properties known to be associated with the disordered status. Therefore, we hypothesized that the central acidic and basic regions are IDRs and contribute to regulate the RNA binding activity of the structured RNA binding domain, CTD. Computational prediction of the disordered status by POODLE-I (http://mbs.cbrc.jp/poodle/poodle-i.html) (31) showed that the central region of B23 is IDR (Figure 2A). To confirm this prediction, we performed CD spectrum analyses with His-tagged B23.1-ΔC3 harboring the N-terminal oligomerization domain, B23.1-ΔN, and B23.1-CR1 harboring bIDR (amino acids 1–119, 120–294 and 189–259, respectively) (Figure 2B). Consistent with the reported structural data demonstrating that the oligomerization domain of B23.1 consists of β-sheets (32), the spectrum recorded for His-B23.1-ΔC3 showed a negative peak at ∼215 nm (green line). His-B23.1-CR1 showed a typical random-coil pattern peaking at 195 nm (blue line), as previously reported (25). The spectrum recorded for His-B23.1 -ΔN showed negative peaks at ∼222 and 200 nm (purple line). Because negative peaks at 222 and 208 nm and that at 200 nm are indicative of α-helix and random-coil structure, respectively, we inferred that His-B23.1 -ΔN contained both α-helix and random-coil structures. Consistent with previous nuclear magnetic resonance studies (24), the spectrum of B23.1-CR1.5 (amino acids 242–294) peaked at ∼222 and 208 nm (Figure 2C, light purple line). When the sequences adjacent to CTD were elongated (His-B23.1-CR and His-B23.1 -ΔN), the peak at 208 nm shifted to ∼200 nm. In addition, the spectrum of His-B23.2 -ΔN (acidic and basic regions with a short portion of CTD) showed a negative peak at 200 nm (Figure 2D, red purple line). These results indicated that the central region of B23 containing acidic and basic regions was disordered in solution. To exclude the possible contribution of His-tag to the B23 protein structure, we prepared non-tagged B23.1, B23-CR1, B23.1 -ΔN and B23.2 -ΔN and compared their spectra with those of His-tagged proteins (Supplementary Figure S4). In all cases, the overall pattern of the spectra was similar, although the peak at ∼200–210 nm of B23.1, B23-CR.1, B23.1-ΔN was slightly shifted to the left when His-tag was added. Thus, we concluded that His-tag did not significantly affect B23 protein structure.

### bIDR and CTD of B23.1 associate with RNA

**Figure 3.**
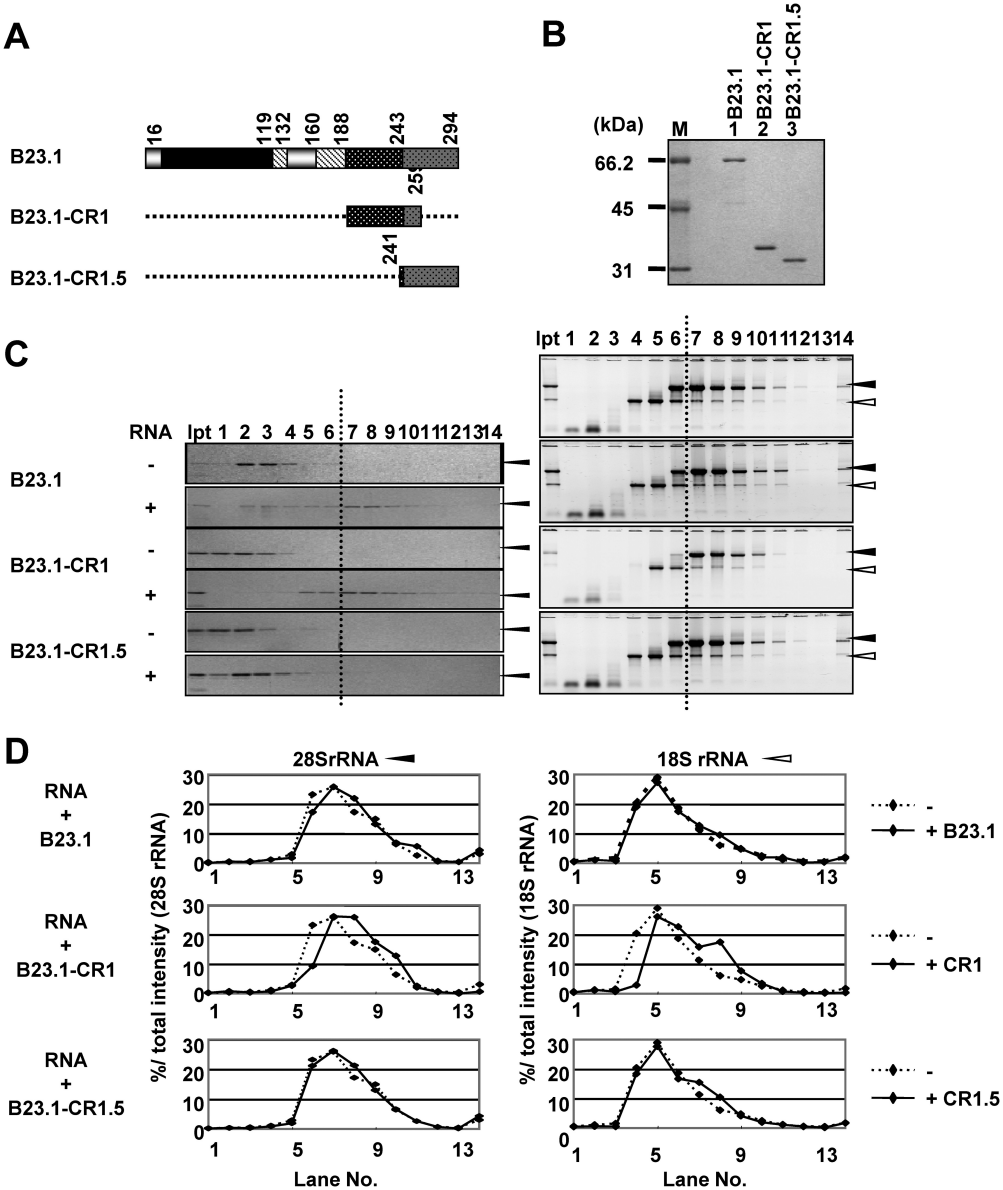
The RNA binding activity of bIDR and CTD of B23.1. (**A**) Schematic representation of B23.1 and mutants. The patterns in the scheme are as defined in Figure 1A. (**B**) Recombinant GST-tagged B23.1 and its mutant proteins were analyzed by SDS–PAGE followed by CBB staining. Lane M indicates molecular markers. (**C**) Sucrose density gradient centrifugation assays. The RNA binding activities of GST-tagged B23.1 and mutant proteins were examined by Sucrose gradient centrifugation as in Figure 1E. Proteins in fractions were analyzed by SDS–PAGE and visualized by CBB staining (left panels). RNA alone (right top panel) and RNAs preincubated with B23 proteins were purified from each fraction, separated on 1% denatured agarose gels and visualized by Gel Red staining (right panels). (**D**) Distribution pattern of 28S and 18S rRNAs. The amounts of 28S (left panels) and 18S rRNAs (right panels) in each fraction in Figure 3C right panels relative to total 28S and 18S rRNAs were calculated and shown in graphs. Dashed lines in each graph indicate the sucrose gradient results of RNAs in the absence of proteins (Figure 3C, top right panel).

To address the function of bIDR of B23, we ivestigated the RNA binding activity of this region. Recent studies indicated that in addition to CTD of B23.1, a part of bIDR was required for its stable G-quadruplex DNA binding activity (24). These results raised the possibility that bIDR is also involved in the RNA binding activity of B23. To test this hypothesis, GST-tagged B23.1-CR1 and B23.1-CR1.5 were purified (Figure 3A and B) and examined for RNA binding activity using sucrose density gradient centrifugation assays. In the absence of RNA, GST-B23.1 and mutant proteins were recovered in low-density fractions (Figure 3C, lanes 1–4). When total RNA was subjected to a sucrose density gradient centrifugation assay, 28S and 18S rRNAs were recovered in fractions 6–10 and 4–9, respectively (Figure 3C, right top panel). GST-B23.1 preincubated with total RNAs was recovered in the fractions yielding 28S rRNA (Figure 3C, right panel). Consistent with this result, the fractionation pattern of 28S rRNA but not of 18S rRNA shifted slightly to the bottom (Figure 3D, top graphs), suggesting that B23.1 preferentially associated with 28S rRNA. When GST-B23.1-CR1 was preincubated with RNA, its distribution pattern shifted to high-density fractions (Figure 3C, left panel, lanes 5–10). In contrast, GST-B23.1-CR1.5 was not clearly cofractionated with rRNAs. Consistent with this result, GST pull-down assays showed that GST-B23.1-CR1 efficiently precipitated both 18S and 28S rRNA but GST-B23.1-CR1.5 and GST did so inefficiently (data not shown). These results indicated that bIDR had potential RNA binding activity and that CTD alone could not efficiently associate with rRNAs. Fractionation patterns of both 28S and 18S rRNAs showed shifts to higher-density fractions when incubated with the GST-B23.1-CR1 protein (Figure 3D). In addition, 28S rRNA preincubated with CR1 shifted to higher-density fractions than that preincubated with wild-type B23.1. On the basis of these results, we predicted that bIDR of B23.1 strongly associates with RNA with low specificity.

B23.1 was recovered primarily in the 28S rRNA peak fractions, but we did not know whether B23.1 directly binds 28S rRNA. To investigate the same, we examined the RNAs contained in the 28S peak fractions. Total RNAs were fractionated by sucrose density gradient centrifugation assay, and the RNAs were separated by agarose gel and polyacrylamide gel electrophoresis (Supplementary Figure S5A). 18S and 28S rRNAs were recovered in fractions 4–9 and 6–10, respectively. In addition, 5.8S rRNA and a small but distinct level of 5S rRNA clearly cofractionated with 28S rRNA. To test whether B23.1 directly binds to 28S but not to 18S rRNA, both 28S and 18S rRNAs were purified. 18S and 28S rRNAs alone were primarily found in fractions 4–6 and 7–9, respectively. We next examined the 28S and 18S rRNA binding activity of B23.1 by sucrose density gradient centrifugation assay (Supplementary Figure S5B and C). Surprisingly, we found that GST-B23.1 was cofractionated with both purified 18S and 28S rRNAs. However, when total RNAs were mixed with purified 18S rRNA and examined for B23.1 binding, GST-B23.1 recovery in the 18S rRNA peak fractions (fractions 4–7) decreased and those in the 28 S rRNA peak fractions (fractions 7–10) increased. These results suggest that B23.1 has some preference for RNA structure or sequence found in RNAs in the 28 S peak fractions such as 28 S and 5.8 S rRNAs.

### Phosphorylation of bIDR contributes to the inactivation of B23.1 RNA binding activity

**Figure 4.**
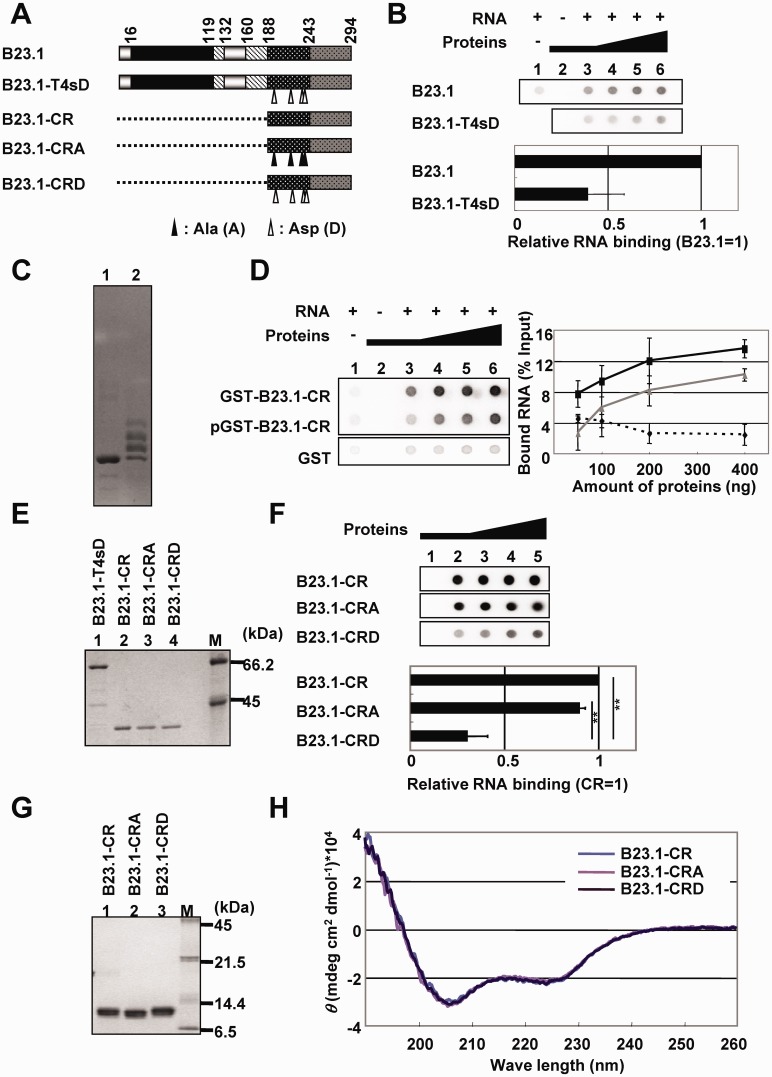
The effect of mitotic phosphorylation on the RNA binding activity of bIDR. (**A**) Schematic representation of B23.1 and unphosphorylatable or phospho-mimetic CR mutants. B23.1-T4sD is a mutant in which threonine residues shown by arrowheads are replaced with aspartic acid (D), and B23.1-CRA and B23.1-CRD are B23.1-CR mutants in which the same residues are replaced with alanine (A) and aspartic acid (D), respectively. (**B**) RNA binding activity of B23.1 and phospho-mimetic mutant. GST-B23.1 and GST-B23.1-T4sD (50, 100, 200 and 400 ng for lanes 3–6, respectively) were mixed with (lanes 3–6) or without (lane 2) ^32^P-labeled total RNA (10 ng). RNA alone was also examined (lane 1). The intensity of each spot was analyzed by FLA7000, and the RNA binding activity obtained with the same amount of B23 proteins were calculated relative to that of B23.1 (1.0). The relative RNA binding activity at each protein amount was averaged. Means ± SD obtained from twice of duplicate independent experiments are shown (bottom panel). (**C**) Phosphorylation of GST-B23-CR. GST-B23.1-CR was incubated with mitotic extracts in the absence (lane 1) or presence (lane 2) of 3 mM ATP. The incubated proteins were analyzed by phos-tag SDS–PAGE and visualized by CBB staining. (**D**) The RNA binding activity of GST, unphosphorylated GST-B23.1-CR and phosphorylated GST-B23.1-CR (pGST-B23.1-CR) were examined for filter binding assays as in (B). The bound RNA for each amounts of GST (dashed line), GST-B23.1-CR (black line) and GST-B23.1-CRD (gray line) were plotted and shown in the right panel. (**E**) Recombinant GST-tagged B23 proteins were analyzed by SDS–PAGE followed by CBB staining. Lane M indicates molecular markers. (**F**) RNA binding activity of B23.1-CR mutants. Filter binding assays were carried out as described in (B) using GST-B23.1-CR, -CRA and -CRD. The amount of RNA retained on the membrane with GST-B23.1-CR is set as 1.0. Statistical *P*-values were calculated by *t*-tests and indicated with **P < 0.01. (**G**) Recombinant His-tagged B23.1-CR, -CRA and -CRD were analyzed by SDS–PAGE and visualized by CBB staining. Lane M indicates molecular markers. (H) CD spectral analysis of B23.1-CR, B23.1-CRA and B23.1-CRD. CD spectra of B23.1-CR (blue gray), B23.1-CRA (light purple) and B23.1-CRD (purple) (5 µM) were analyzed.

Previously, we have demonstrated that the phosphorylation of B23 by cdc2/cyclin B kinase reduces its RNA binding activity (26). The phosphorylation sites, threonine 199, 219, 234 and 237, are located in bIDR. Consistent with previous results (28), the phosphomimetic mutant of four threonine residues (T4sD) (Figure 4A and B) showed significantly lower RNA binding activity than wild-type B23.1 (Figure 4B). Therefore, we hypothesized that mitotic phosphorylation regulated the RNA binding activity of bIDR. To test this hypothesis, B23.1-CR was phosphorylated by mitotic extracts prepared from HeLa cells, and the RNA binding activity of phosphorylated protein was assessed (Figure 4C and D). Phosphorylation status of the protein was examined by Mn^2+^-phos-tag® SDS–PAGE (compare lanes 1 and 2 in Figure 4C). Although the CR protein was not completely phosphorylated, the RNA binding activity of phosphorylated GST-B23.1-CR was lower than that of untreated GST-B23.1-CR (Figure 4D). To gain clearer insight into this point, phosphomimetic (CRD) and unphosphorylatable (CRA) mutants of GST-tagged B23.1-CR, in which threonine (T) residues were replaced with aspartic acid (D) and alanine (A) residues, respectively, were expressed and examined for the RNA binding activity using filter binding assay (Figure 4E and F). Labeled RNA was retained on the membrane when incubated with GST-B23.1-CR and GST-B23.1-CRA mutant proteins. However, the activity of GST-B23.1-CRD was significantly lower than that of GST-B23.1-CR and GST-B23.1-CRA (∼30% of GST-B23.1-CR). The GST pull-down assay also indicated that the 28 S and 18 S rRNA binding activities of GST-B23.1-CRD were ∼35 and 25% of those of GST-B23.1-CR (Supplementary Figure S6). CD spectral analyses of the three mutant proteins showed peaks at ∼222 and 208 nm, the typical pattern of α-helix (Figure 4G and H), suggesting that the phosphomimetic mutation at the phosphorylation sites does not affect the C-terminal helical structure. Moreover, in the presence of a low concentration of RNA, the CD spectrum of bIDR (CR1, see Figure 3B) was not significantly changed (Supplementary Figure S7). This finding suggests that a specific structure of bIDR is not induced on binding to RNA.

**Figure 5.**
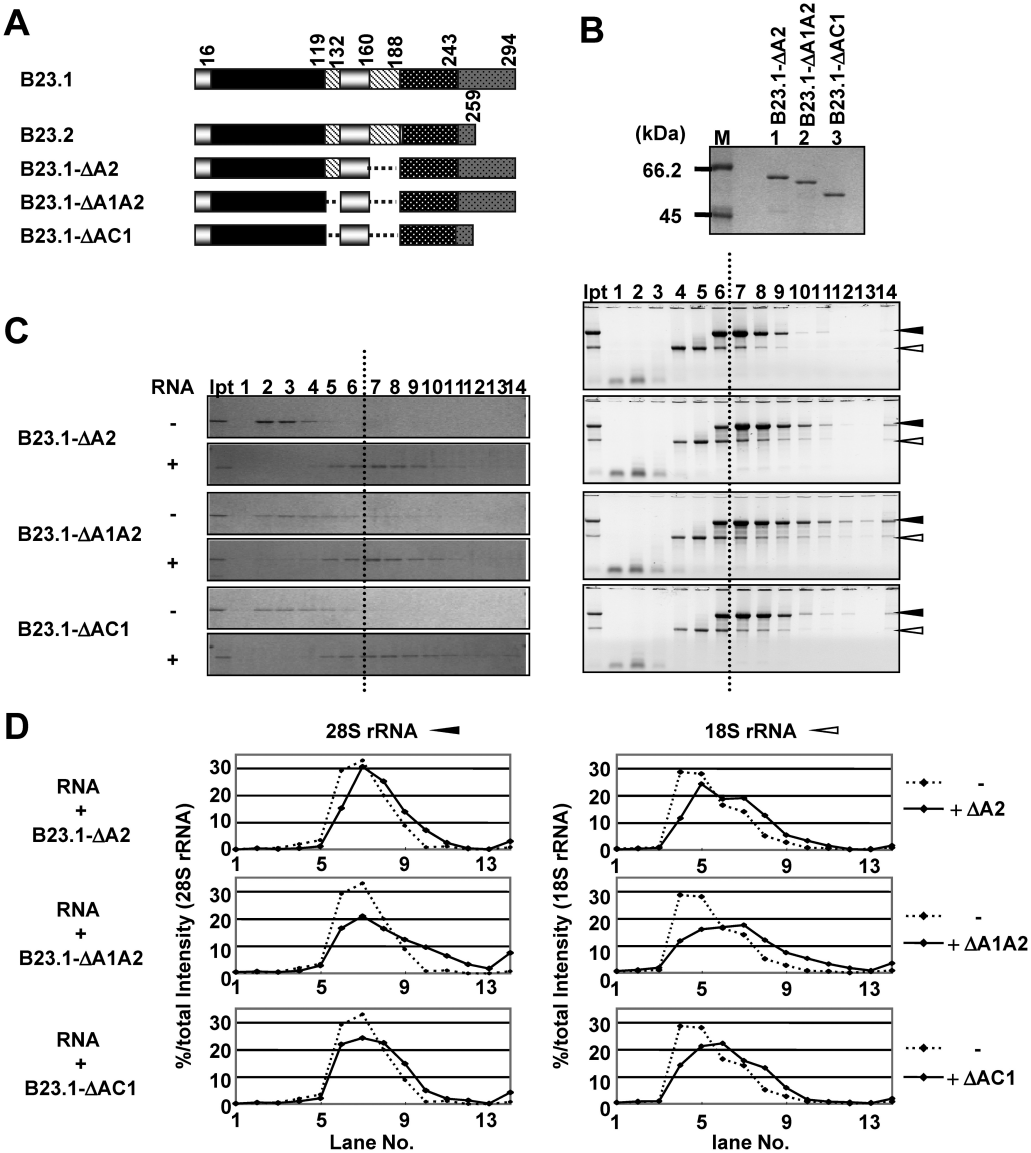
aIDR is required for the proper RNA binding of B23. (**A**) Schematic representation of B23.1 and deletion mutants. (**B**) Recombinant GST-tagged B23 mutant proteins were analyzed by SDS–PAGE and visualized by CBB staining. Lane M indicates molecular markers. (**C**) Sucrose density gradient centrifugation assays. Total RNAs (10 µg) preincubated in the absence (top panel) or presence of GST-tagged B23 proteins (5 µg) were loaded on a 15–40% sucrose gradient. Proteins in fractions collected from the top were analyzed by SDS–PAGE and visualized by CBB staining (left panels). RNAs recovered in each fraction were analyzed as in Figure 3C (right panels). Right top panel is data for RNA alone. (**D**) Distribution patterns of 28S and 18S rRNAs. The intensity of 28 and 18S RNAs in C, right panels were scanned and analyzed by CS analyzer (ATTO) and graphically represented. Dashed lines are the sucrose gradient results of RNA alone.

bIDR of B23 showed strong RNA binding activity, whereas B23.2, containing identical amino acid sequences in this region, did not efficiently associate with RNA, suggesting that the RNA binding activity of bIDR is modulated by full-length B23 protein. Because B23 has an aIDR, it is likely that negative charge of the aIDR neutralizes the positive charge of bIDR. To examine this possibility, we studied the effect of aIDR deletion on B23 RNA binding activity using B23.1 -ΔA2, B23.1 -ΔA1A2 and B23.1-ΔAC1 (Figure 5A). Sucrose density gradient centrifugation assays clearly showed that the aIDR mutants associated with RNA (fractions 5–10). The fractionation pattern of 28S and 18S rRNAs indicated that the aIDR mutants associated with both rRNAs. The fraction shifts in fractions of both rRNAs incubated with GST-B23.1-ΔA1A2 were more prominent than those of RNAs incubated with the B23.1-CR1 mutant (see Figure 3C and D), possibly due to the oligomer formation of GST-B23.1-ΔA1A2. In addition, both 28S and 18S rRNAs incubated with GST-B23.1-ΔA1A2 were recovered in higher-density fractions than those incubated with GST-B23.1-ΔA2 (Figure 5C and D).

Furthermore, GST-B23.1-ΔAC1 associated with RNAs, as did GST-B23.1-ΔA1A2, indicating that CTD was not required for RNA binding when aIDR was deleted. From these results, we concluded that the RNA binding activity of bIDR is negatively regulated by aIDR and that aIDR confers RNA binding specificity on B23.1.

**Figure 6.**
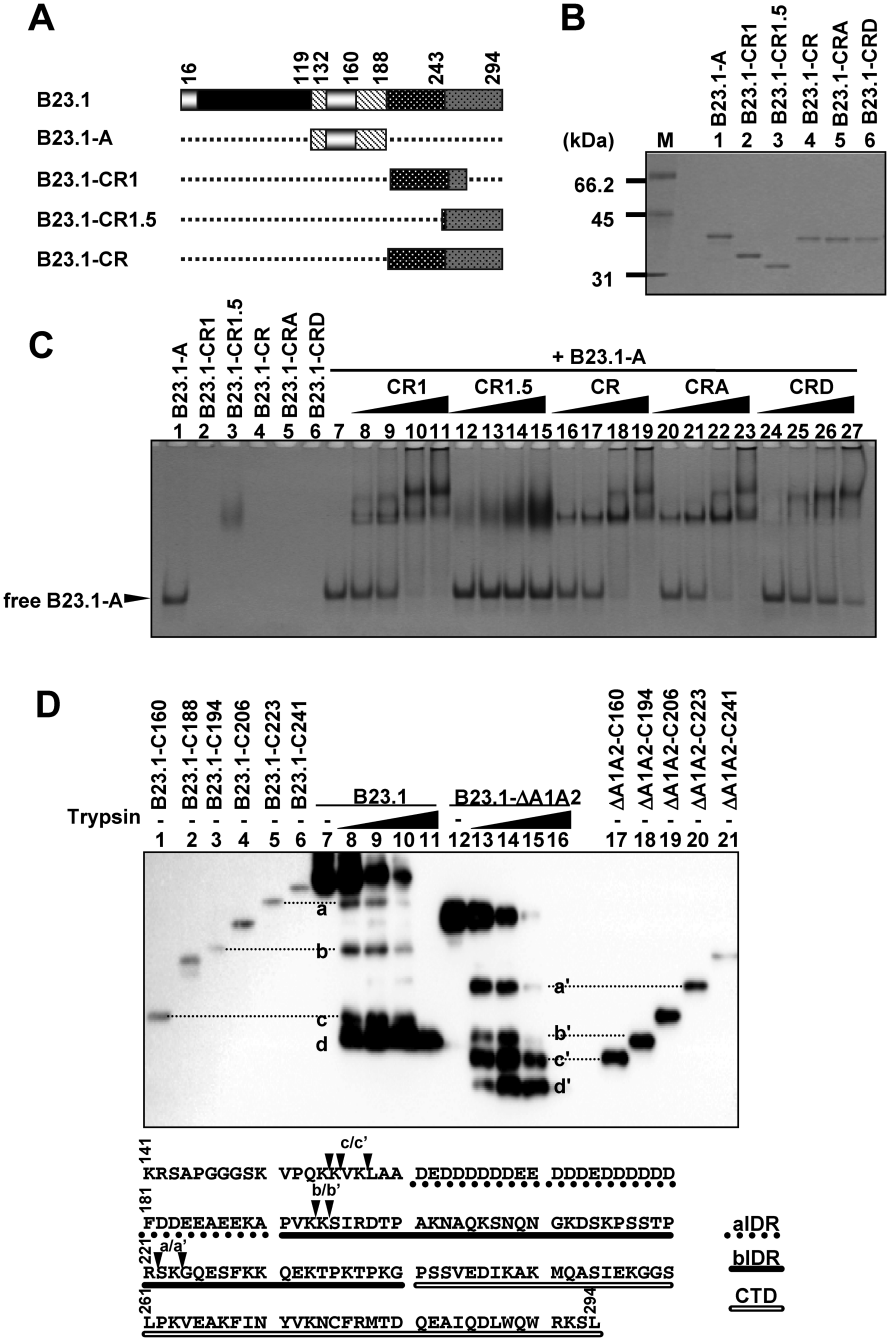
Association between two IDRs. (**A**) Schematic representation of B23 and deletion mutants. (**B**) Recombinant GST-tagged B23 mutant proteins were analyzed by SDS–PAGE and visualized by CBB staining. Lane M indicates molecular markers. (**C**) Mobility shift assays. GST-B23.1-A (0.5 µg) mixed with increasing amounts of GST-B23.1-CR1 (lanes 8–11), GST-B23.1-CR1.5 (lanes 12–15), GST-B23.1-CR (lane 16–19), GST-B23.1-CRA (lanes 20–23) and GST-B23.1-CRD (lanes 24–27) (0.5, 1.0, 2.0, and 4.0 µg) were incubated for 30 min, separated by 6% native PAGE in 0.5× TBE, and visualized by CBB staining. (**D**) Trypsin digestion of B23.1 and B23.1 -ΔA1A2. His-B23.1 (lanes 7–11) and His-B23.1 -ΔA1A2 (lanes 12–16) were incubated with trypsin (0, 2, 8, 20 and 40 ng) for 2 min at 37°C, and digested proteins were separated on SDS–PAGE and detected with western blotting using anti-His-tag antibody. As references for the digestion sites, a series of C-terminal deletion mutants (lanes 1–6 and 17–21) were also separated by the same SDS–PAGE. C160, C188, C194, C206, C223 and C241 indicate B23.1 or B23.1 -ΔA1A2 proteins containing amino acids 1–160, –188, –194, –206, –223 and –241, respectively. Amino acid numbers are from the full-length protein. The digested peptides of His-B23.1 and His-B23.1-ΔA1A2 are indicated by a–d and a'–d', respectively. B23.1 amino acids 141–294 are shown at the bottom. Putative digestion sites (a/a’, b/b’ and c/c’) are mapped by black arrowheads. Dashed underline, black underline and double underline indicate aIDR, bIDR and CTD, respectively.

We subsequently tested whether the two regions associated with each other. GST-tagged B23-A (amino acids 120–188) was mixed with increasing amounts of B23.1-CR1, B23.1-CR1.5, B23.1-CR, B23.1-CRA or B23.1-CRD (Figure 6A and B), and the mixtures were loaded on native PAGE (Figure 6C). GST-B23.1-A migrated toward the cathode faster than other mutants because of its negative charge (the theoretical pI of aIDR is 3.6), whereas GST-B23.1-CR1, -CR, -CRA and -CRD did not enter the gel (pI; 10.2, 10.0, 10.0 and 9.7, respectively). When GST-B23.1-A was incubated with increasing amounts of GST-B23.1-CR1, -CR or -CRA, free form of GST-B23.1-A decreased and low-mobility bands appeared (lanes 8–11, 16–19 and 20–23). In contrast, addition of GST-B23.1-CR1.5 did not change the mobility of GST-B23.1-A (lanes 12–15). Furthermore, the binding activity of GST-B23.1-A with GST-B23.1-CRD was lower than that of GST-B23.1-CR1, -CR and -CRA (lanes 24–27). In addition, the binding activity of GST-B23.1-CR phosphorylated by mitotic extracts to GST-B23.1-A was weaker than that of the unphosphorylated protein (Supplementary Figure S8). These results indicate that aIDR regulates the non-specific RNA binding activity of bIDR through direct association. Moreover, phosphorylation in bIDR diminished the RNA binding activity and the interaction with aIDR. To assess the association between aIDR and bIDR in full-length B23.1, we tested the sensitivity of His-tagged B23.1 and B23.1-ΔA1A2 toward trypsin (Figure 6D). Digested peptides were detected by anti-His-tag antibody to map digested sites. A series of C-terminal deletion mutants (His-B23.1-C160, -C188, -C194, -C206, -C224 and -C241, and a corresponding set of mutants His-B23.1-ΔA1A2-C160, -C194, -C206, -C224 and -C241) was used as size markers. On treatment with trypsin, full-length B23.1 proteins decreased, whereas four digested bands were detected by anti-His tag antibody. The digested bands d and d' were lower than those of His-B23.1-C160 (amino acids 1–160) and His-B23.1-ΔA1A2-C160, suggesting that the bands d/d' correspond to the product digested at amino acids 140–150. The sizes of bands a/a', b/b' and c/c' were similar to those of the C223, C194 and C160 mutants, respectively. The intensities of the bands a, b and c relative to full-length proteins were lower than those of a', b' and c' relative to full-length His-B23.1-ΔA1A2. These results strongly suggest that bIDR and the region 133–160 of B23.1 are susceptible to trypsin degradation when aIDR is deleted. These results support the idea that aIDR of B23.1 regulates its bIDR by direct binding in its native full-length form.

### Inter- and intra-molecular interaction between IDRs in B23

**Figure 7.**
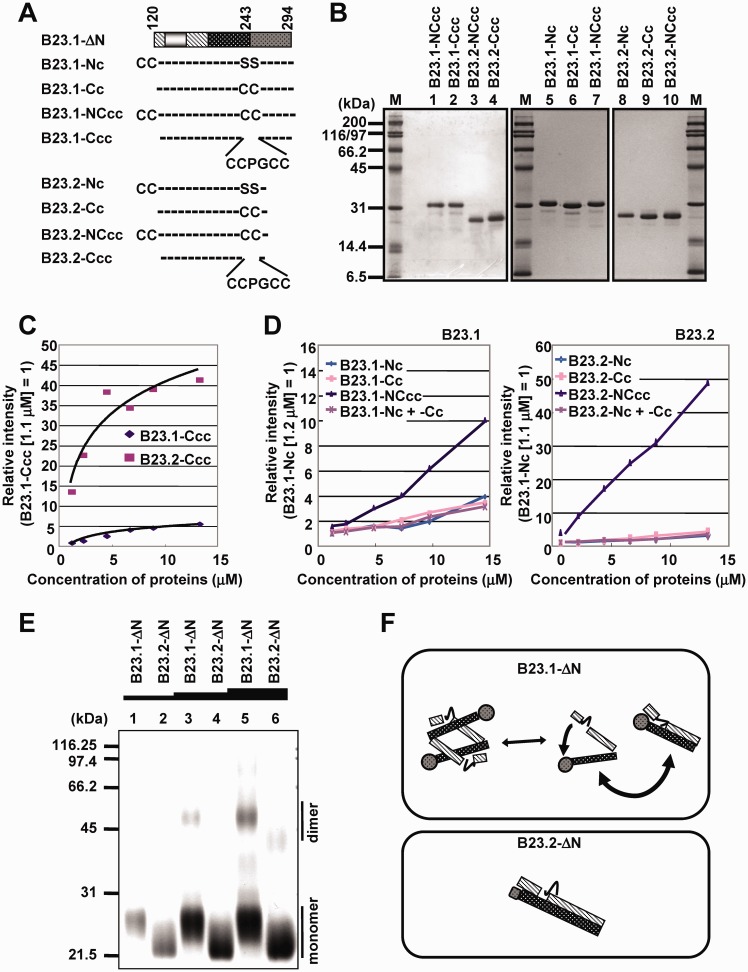
Intra- and inter-molecular interaction between two IDRs of B23.1 and B23.2. (**A**) Schematic representation of B23 mutants. B23.1/B23.2-ΔN and their Cys–Cys-tagged mutants are schematically represented. The patterns in the scheme of B23.1-ΔN are as defined in Figure 1A. N-terminal Cys–Cys tag was represented as CC- (Cys–Cys-). C-terminal Cys-Cys-tag was introduced by replacement of serines [S] 242 and 243 with cysteines [C], and represented as -CC-. Tetracysteine-tag (Cys–Cys–Pro–Gly–Cys–Cys, CCPGCC) was introduced into the same site with -CC-. (**B**) Purified proteins. The Cys-Cys-tagged mutant proteins (200 ng) as indicated at the top of each lane were separated by SDS–PAGE and visualized with CBB staining. (**C**) FlAsH labeling of B23.1-Ccc and B23.2-Ccc. His-tagged B23.1-Ccc (diamond) or B23.2-Ccc (square) (1.1, 2.2, 4.4, 6.6, 8.8 and 13.2 µM) was incubated with 10 µM of FlAsH-EDT_2_ for 30 min at room temperature, and the fluorescence intensity was recorded with Varioskan. (**D**) FlAsH labeling of B23 mutants. His-tagged B23-Nc (light blue), B23-Cc (pink), B23-NCcc (purple) and mixture of B23-Nc and B23-Cc (light purple line) [1.2, 2.4, 4.9, 7.3, 9.7 and 14.4 µM (B23.1), or 1.1, 2.2, 4.4, 6.6, 8.8 and 13.2 µM (B23.2)] were examined for FlAsH labeling assay as in (B). (**E**) Cross-link assay. His-tagged B23.1-ΔN and B23.2-ΔN (1.5, 3.0 and 4.5 µM) (lanes 1 and 2, 3 and 4 and 5 and 6) were incubated in the presence of 0.1% Glutaraldehyde for 10 min, separated on SDS–PAGE, and visualized by CBB staining. Positions of size markers are indicated at the left of the panel. (**F**) A model for B23.1-ΔN and B23.2-ΔN structures. The globular structure of CTD is illustrated by circle, and stripes and black with white dots bars indicate aIDR and bIDR, respectively.

bIDR of B23.1 was essential for efficient RNA binding activity, although the RNA binding activity of the same amino acid sequences (bIDR) in B23.2 was suppressed. Therefore, we hypothesized that the interaction mode of acidic and bIDRs in B23.1 was different from that in B23.2. In addition, it was unclear whether the interaction between the two IDRs was inter- or intra-molecular. To address these points, we tested the interaction between the two IDRs of B23 proteins using FlAsH labeling (Figure 7A–D). FlAsH, a biarsenical dye, fluoresces on binding to the thiol moieties of cysteines (Cys) in the tetracysteine (Cys–Cys–Xaa–Xaa–Cys–Cys) (33). If tetracysteine is separated into two Cys–Cys pairs, FlAsH fluoresces only when two Cys–Cys pairs are in close proximity (6–8 Å) (33,34). For this assay, we constructed a series of B23.1-ΔN and B23.2-ΔN mutants containing Cys–Cys tags (Figure 7A). One Cys–Cys tag was introduced at the N-terminal of aIDR (B23-Nc and B23.2-Nc), and the other was introduced at positions 242 and 243 (both serines) located at the border between bIDR and CTD (B23.1-Cc and B23.2-Cc). The ‘-NCcc’ proteins have two Cys–Cys tags at both sites. B23.1-Ccc and B23.2-Ccc, in which serines at 242 and 243 were replaced with the known FlAsH binding tetracysteine sequence (Cys–Cys–Pro–Gly–Cys–Cys) (35), were used as positive controls (Figure 7A and B). When increasing amounts of B23.2-Ccc were mixed with FlAsH, fluorescence was detected in a dose-dependent manner (Figure 7C). Fluorescence was also detected when B23.1-Ccc was examined. However, the fluorescence intensity of B23.1-Ccc was only ∼10% of that of B23.2-Ccc (*P* < 0.0005). This result suggests that CTD of B23.1 restricted the access of FlAsH molecule to the tetracysteine sequence located between bIDR and CTD. When increasing amounts of B23.1-Nc, B23.1-Cc, B23.2-Nc and B23.2-Cc harboring a single Cys–Cys pair were mixed with FlAsH, very weak fluorescence was detected (Figure 7D). A mixture of B23.1-Nc and B23.1-Cc or of B23.2-Nc and B23.2-Cc also showed weak fluorescence (Figure 7D). In contrast, in case of B23.1-NCcc and B23.2-NCcc harboring two Cys–Cys pairs, fluorescent signals were dependent on protein concentration. These results suggest that both B23.1-ΔN and B23.2-ΔN bent to form a hairpin-like structure, and the two Cys–Cys pairs in one molecule were located close to each other. Although we could not detect inter-molecular interaction using FlAsH labeling, it was possible that it could not be detected because of the distance between the two Cys–Cys pairs. To examine this possibility, chemical cross-link assays were performed (Figure 7E). His-tagged B23.1-ΔN and B23.2-ΔN were cross-linked with 0.1% glutaraldehyde and separated using SDS–PAGE, followed by CBB staining. Both His-B23.1-ΔN and His-B23.2-ΔN were found to be monomers at low protein concentrations because of the absence of the N-terminal oligomerization domain (Figure 7E, lanes 1 and 2). Bands corresponding to dimers appeared when His-B23.1-ΔN protein concentration was increased, whereas the dimer formation of His-B23.2-ΔN was inefficient (Figure 7E, lanes 5 and 6). This result suggests that inter-molecular interaction between aIDR and bIDR in B23.1 occurs more frequently compared with that in B23.2. Monomer forms of these proteins were likely to form intra-molecular interactions as shown by FlAsH labeling assays (Figure 7D). Therefore, we conclude that aIDR and bIDR of both B23.1-ΔN and B23.2-ΔN interact intra-molecularly, and that B23.1-ΔN also has the potential to form inter-molecular interactions (Figure 7F).

## DISCUSSION

In this study, we investigated the molecular mechanism by which B23.1 associates with RNA. CD spectral analysis revealed that the central acidic and basic regions of B23 were intrinsically disordered (Figure 2). Our results suggest that B23.1 recognizes RNA through two fragments, bIDR and CTD, and that aIDR modulates the non-specific RNA binding activity of bIDR, allowing B23 CTD to recognize and interact with target RNAs. In the absence of CTD, the non-specific RNA binding activity of bIDR was inhibited by aIDR, indicating that B23.2 inefficiently associates with RNA. This model is based on three findings: (i) bIDR binds directly to RNAs (Figures 3 and 4); (ii) the RNA binding activity of bIDR of B23 is regulated by its aIDR (Figures 5 and 6); and (iii) the association mode of the acidic and bIDRs is different between B23.1 and B23.2 (Figure 7). The regulatory functions of IDRs could be biologically important because bIDR deletion (B23.3) increased and aIDR deletion (B23.1-ΔA1A2) decreased cellular mobility of B23 (Figure 1C and Supplementary Figure S9).

CTD of B23.1 is known as the DNA and RNA binding domain. Recent studies have shown that the small groove formed by the first two α-helices in CTD recognizes the phosphate group of the G-quadruplex DNA (24). Basic amino acids located at the surface of CTD allow electrostatic interaction with DNA. The affinity of CTD alone to G-quadruplex DNA was weak (*K_D_* > 1 × 10^−^^5 ^M), but it increased on addition of flanking sequences (*K_D_* was ∼2 × 10^−^^6 ^M) (23). Consistent with this, the RNA binding activity of CTD was low, and full bIDR was required for efficient RNA binding by CTD. In the complex with G-quadruplex DNA, bIDR does not directly associate with DNA (24), and the role of bIDR is inferred to stabilize the structure of CTD (25). In this study, we found that bIDR alone could bind to RNA (Figure 3), and B23.3 lacking most of bIDR showed significantly lower RNA binding activity than B23.1 (Figure 1). These results indicate that bIDR is required for the RNA binding activity of B23.1. This activity of bIDR is likely to help CTD to identify and recognize target RNA sequences or structures by direct binding to RNA. Considering that CTD preferentially binds to structured RNA or DNA, bIDR may stabilize the binding by interacting with flanking RNA/DNA sequences. The RNA recognition mode of B23.1 is extremely similar to that used by several transcription factors and the linker histone H1. For example, histone H1 binds to the nucleosome at the DNA entry and exit sites through its central globular domain (36,37). In human cells, the absence of the basic C-terminal ID region of histone H1 significantly decreases the fluorescent recovery time after photobleaching (18.7 ± 5.7 s for the wild-type and 0.49 ± 0.11 s for the deletion mutant) (38). It is suggested that the C-terminal ID region confers the stable nucleosome binding activity on histone H1 by interacting with the linker DNA region between the nucleosomes.

Here we show that full-length B23.1 seems to bind preferentially to 28S rRNA through its CTD. However, which sequence or structure in the RNA is recognized by B23 is currently unknown. It has been reported that B23 binds to G-quadruplex DNAs (39). Because G-rich RNAs also form G-quadruplex structures, G-rich RNA regions are possibly those recognized by B23. Both 18S and 28S rRNAs have G-rich sequences predicted to form G-quadruplex structures (data not shown), and both purified rRNAs are associated with B23 (Supplementary Figure S5B). Of interest, 18S rRNA binding of B23 decreased on total RNA addition. These results suggest that the RNA structure or sequence in 18S rRNA targeted by B23 is disrupted or hindered by other RNA species. It is also possible that B23 associates with 28S rRNA complexed with other RNA species with much higher affinity than that with18S rRNA or 28S rRNA alone. It is well established that 28S rRNA is strongly bound to 5.8S rRNAs by hydrogen bonds (40,41). Thus, double-stranded region of the 28S–5.8S rRNAs may be recognized by B23.

B23.2 lacking CTD did not efficiently associate with RNA (Figure 1). We assume that the RNA binding activity of bIDR of B23.2 was completely inhibited by aIDR possibly because of the hairpin-like structure formation (see Figure 7F). Intra-molecular interaction between these regions may be physically hindered by CTD in full-length B23.1, and this interaction becomes more dynamic and inter-molecular between adjacent molecules. This hypothesis was supported by the observation that the accessibility of the FlAsH fluorophore to the site between bIDR and CTD was restricted in B23.1-Ccc (Figure 7C). Previously, we have demonstrated that B23.1–B23.2 and B23.1–NPM3 hetero-oligomers show lower RNA binding activity than that by the B23.1 homo-oligomer (26,30). B23.2 or NPM3 incorporation in the B23.1 oligomer may decrease the chance to establish inter-molecular interactions between adjacent molecules of B23.1, possibly inhibiting bIDR function by aIDR. It should also be mentioned that the stochastic interaction between the two IDRs increases depending on protein concentration. Because B23 forms a pentamer or decamer in solution, the local concentration of IDRs is increased, and this may allow inter-molecular interactions in B23.1 homo-oligomers. Given that two B23 pentamers form a decamer in a head-to-head fashion (20,32), two possible interaction modes can be considered: One is that bIDR associates with aIDR of a neighboring protein in a pentamer and the other is that bIDR associates with aIDR in the other pentamer (see Supplementary Figure S10).

B23 is known to be subjected to various post-translational modifications including phosphorylation, acetylation and sumoylation (26,42–47). Our results clearly explained why mitotic phosphorylation by cdc2/cyclin B kinase at bIDR decreases the RNA binding activity. It has been reported that lysines at positions 257 and 267 of B23.1 located at the surface of CTD (24) are acetylated by p300 and that these acetylations induce the translocation of B23.1 from the nucleolus to the nucleoplasm (46). Because the G-quadruplex DNA and RNA binding activity of B23.1 strongly affect its nucleolar localization (28,39), it is likely that acetylation reduces the DNA/RNA binding activity by neutralizing the positive charge required for its contact with target sequences. Ser125 located in aIDR is known to be phosphorylated by casein kinase 2 (43). Previous studies have demonstrated that phosphorylation of Ser125 decreases the RNA binding activity (28) and nucleolar retention time of B23.1 (48). We hypothesize that the increased negative charge in aIDR by phosphorylation augments its affinity to bIDR, resulting in the decreased DNA/RNA binding activity of bIDR. In summary, post-translational modifications in aIDR, bIDR and CTD all affect the DNA/RNA binding activity of B23.1. Regulation of B23.1 functions by post-translational modification is mechanistically explained by the inter- or intra-molecular interactions between the two IDRs. Moreover, because these regulatory regions are IDRs, the accessible surface area of modification enzymes is believed to be larger than the structured domains, thereby rapidly regulating the functions of B23.1. Thus, these modifications contribute to fine-tuning the B23.1 functions to control proper cell growth and proliferation.

B23 associates with various proteins in cells, such as p14ARF (49), histones (27,50) and p53 (51,52), through its N-terminal domain, aIDR and bIDR, respectively. Because the RNA binding activity of B23.1 is regulated by the association between acidic and bIDRs, it is presumed that when bIDR of B23.1 is occupied by RNA or aIDR is occupied by histones, the association of the two IDRs is disturbed. These interactions with other molecules may reciprocally increase the affinity of these regions for target molecules. Therefore, it is likely that B23.1 tethered by RNA molecules on chromatin around the *rRNA* gene (28) functions as an active histone chaperone.

## SUPPLEMENTARY DATA

Supplementary Data are available at NAR Online.

## FUNDING

Grant-in-Aid for Scientific Research from the Ministry of Education, Culture, Sports, Science and Technology, Japan [grant numbers 22570132 and 21113005] (to M.O. and K.N.) and Takeda Foundation. NIMS Molecule & Material Synthesis Platform in “Nanotechnology Platform Project” operated by the MEXT, Japan. Funding for open access charge: Grant-in-Aid for Scientific Research from the Ministry of Education, Culture, Sports, Science and Technology, Japan [21113005 to M.O.].

*Conflict of interest statement*. None declared.

## Supplementary Material

Supplementary Data
