# Supplementary material for: Intrinsically disordered regions of nucleophosmin/B23 regulate its RNA binding activity through their inter- and intra-molecular association
Source: Nucleic Acids Res. 2013 Oct 6;42(2):1180–95. doi: 10.1093/nar/gkt897 (PMC3902904; doi:10.1093/nar/gkt897)
Supplement: Supplementary Data [file supp_42_2_1180__index.html]

Intrinsically disordered regions of nucleophosmin/B23 regulate its RNA binding activity through their inter- and intra-molecular association — Intrinsically disordered regions of nucleophosmin/B23 regulate its RNA binding activity through their inter- and intra-molecular association — Supplementary Data 

# Intrinsically disordered regions of nucleophosmin/B23 regulate its RNA binding activity through their inter- and intra-molecular association

## Supplementary Data

files

**Files in this Data Supplement:**

- Supplementary Data - pdf file
