# Supplementary material for: Intrinsically disordered regions of nucleophosmin/B23 regulate its RNA binding activity through their inter- and intra-molecular association
Source: Nucleic Acids Res. 2013 Oct 6;42(2):1180–95. doi: 10.1093/nar/gkt897 (PMC3902904; doi:10.1093/nar/gkt897)

Supplementary materials and methods

Preparation of non-tag recombinant proteins-To construct the vectors for expression of recombinant proteins containing protease recognition sequence, cDNAs were digested from pGEX2T-B23.1-ΔN, - B23.2-ΔN, and B23.1-CR by BamHI, and subcloned into the same sites of pGEX6P. Expression and purification using Glutathione sepharose (GE Healthcare) until elution step were described in the main text. GST-tagged proteins bound to Glutathione sepharose were digested by 16 units of Turbo 3C protease (Accelagen Inc.) at 4°C for over night in a buffer containing (50 mM Tris-HCl [pH 8.0], 150 mM NaCl, and 1 mM EDTA). The supernatant was collected and protease was removed by additional incubation with fleshly prepared Glutathione sepharose at 4°C for 1 hour. The supernatant was dialyzed against appropriate buffers.

The mass spectrometry analysis-For mass spectrometry analysis, purified recombinant proteins were dialyzed against water, added trifluoroacetate (final concentration 0.1% (v/v)) and equal amount of sinapic acid as a matrix, and analyzed by matrix-assisted laser-desorption time-of-flight mass spectrometry (MALDI-TOF-MS) (AXIMA®-TOF^{2}, Shimadzu corporation).

Isolation of 18S and 28S rRNAs-To isolate 18S rRNA and 28S rRNA, total RNA purified from 293T cells was dissolved in a buffer containing (1×MOPS buffer (20 mM MOPS [pH 7.0], 2 mM NaOAc, 1 mM EDTA), and 50% (v/v) formamide) and boiled at 65°C for 5 minutes, and separated on 0.8% agarose in 1×MOPS buffer. 18S rRNA and 28S rRNA were electrically eluted from a piece of gels in dialysis tubes, and purified with phenol chloroform extraction and isopropanol precipitation.

Supplementary Figure legends

Supplementary Figure S1. The mass analysis of recombinant proteins. (A) The masses of non-tagged B23.1-ΔN, B23.2-ΔN, and B23.1-CR, and HIS-tagged B23.1-CR, -CRA, and -CRD were analyzed by MALDI-TOF/MS. The theoretical masses are indicated at the top right of each panel. The peaks of analyzed proteins are shown by red arrowheads. (B) Purities of recombinant proteins used in (A) were analyzed by SDS-PAGE followed by CBB staining. Lane M indicates molecular markers. Data for His-tagged B23.1CR, -CRA, and CRD (lanes 4-6) are same with the data shown in Figure 4C. (C) The bands in each lane in (B) were scanned and analyzed by Image J, and graphically represented. The relative abundance of main band is shown in the graph.

Supplementary Figure S2. Quantification of co-immunoprecipitated RNAs with EF-tagged B23 variants. (A) EGFP-Flag (EF) and EF-B23.1, -B23.2, and -B23.3 transiently expressed in 293T cells were precipitated with anti-FLAG antibody. Input (lanes 1–8, 0.5% and 1.5%) and immunoprecipitated RNAs (lanes 9–16, 5% and 15%) were purified and separated on 1% denaturing agarose gel (Top) and 6% Urea-PAGE (bottom), followed by ethidium bromide and Gel Red staining, respectively. The position of 28S, 18S, 5.8S, and 5S rRNAs are shown by arrowheads. (B) The band intensity of 28S, 5.8S, and 5S rRNAs were analyzed by CS analyzer (ATTO) and graphically represented.

Supplementary Figure S3. The RNA binding activities of GST, non-tag B23.1, and GST-B23.1. (A) GST (5 μg) incubated with or without total RNA (10 μg) and total RNA alone (10 μg) were separated on 15%–40% sucrose gradients. Proteins in each fraction were analyzed by SDS-PAGE and visualized by CBB staining (top panels). RNA alone and RNA pre-incubated with GST were purified from each fraction, separated on 1% denaturing agarose gels, and visualized by ethidium bromide staining (bottom panels). (B) The RNA binding activity of non-tagged and GST-tagged B23.1 (5 μg) were examined with sucrose density gradient centrifugation assays as described in (A). Proteins in each fraction were analyzed by SDS-PAGE and visualized by CBB staining.

Supplementary FigureS4. CD spectral analyses of HIS-tagged and non-tagged B23 proteins. (A) Recombinant proteins used in CD spectral measurements were analyzed by SDS-PAGE followed by CBB staining. (B)–(E) The CD spectra were recorded with HIS-tagged (black) or non-tagged (gray) B23.1 (B); B23.1-CR (C); B23.1-ΔN; (D), and B23.2-ΔN (E) (5 μM).

Supplementary Figure S5. The RNA binding preference of B23.1. (A) Total RNA (10 μg) and isolated 28S rRNA and 18S rRNA (estimated amount (5.8 μg and 2.08 μg for 28S and 18S rRNAs) in 10 μg of total RNA) were separated on 15%–40% sucrose gradients. RNAs were purified and analyzed with 1% denaturing agarose gel (top and bottom two panels) and 6% Urea-PAGE (second panel) followed by ethidium bromide staining. The positions of 28S, 18S, 5.8S, and 5S rRNAs are shown by arrowheads. (B) GST-B23.1 (5 μg) was mixed with isolated 18S rRNA (5 μg), 28S rRNA (5 μg), and the mixture of 18S rRNA (2.08 μg) and total RNA (10 μg) were separated on 15%–40% sucrose gradients. Proteins in each fraction were analyzed with SDS-PAGE followed by CBB staining (upper panels). RNAs were purified and analyzed with 1% denaturing agarose gel (lower panels), followed by ethidium bromide staining. (C) Amounts of GST-B23.1 incubated with purified 18S rRNA (blue) and purified 18S rRNA mixed with total RNA (black) in each fraction of (B) relative to that of input was calculated and shown in graph. The amounts of GST-B23.1 incubated with total RNA (red) were estimated from Supplementary Figure S3B bottom panel.

Supplementary Figure S6. The RNA binding activity of GST, GST-B23.1-CR, -CRA, and -CRD. The RNA binding activity of GST, GST-B23.1-CR, -CRA, and -CRD was examined with GST pull-down assays. GST, GST-B23.1-CR, -CRA, and -CRD (2 μg) were bound to Glutathione sepharose beads, and incubated with total RNA purified from 293T cells (5 μg). After extensive washing with buffer containing 300 mM NaCl, RNAs co-precipitated with GST proteins were purified and 20% of precipitated RNAs for each GST protein and 2.2, 6.7, and 20% of input RNAs were separated by 1% agarose gel electrophoresis (left panels). The intensities of RNA bands were measured by CS analyzer (ATTO) and graphically represented (right panel).

Supplementary Figure S7. The CD spectral analysis of bIDR with RNA. The CD spectra was recorded with total RNA alone (4.8 ng/μl (pink) and 24 ng/μl (red)) (top panel) and HIS-B23.1-CR1 (5 μM) (black) in the absence or presence of total RNA (4.8 ng/μl (pink) and 24 ng/μl (red)) (bottom panel).

Supplementary Figure S8. Association between aIDR and GST-B23.1-CR. (A) GST-B23.1-A (0.2 μg) mixed with increasing amounts of unphosphorylated GST-B23.1-CR (lanes 4–8) and phosphorylated GST-B23.1-CR (pGST-B23.1-CR) (lanes 9–13) (0.1, 0.2, 0.4, 0.8, and 1.6 μg) were incubated for 30 minutes, separated by 6% native PAGE in 0.5×TBE, and visualized by CBB staining. The position of unbound GST-B23.1-A is shown by an arrowhead. (B) The amounts of GST-B23.1-A bound with GST-B23.1-CR (black diamond) and pGST-B23.1-CR (gray square) were calculated by measurement of unbound GST-B23.1-A in (A) using CS analyzer (ATTO), and graphically represented.

Supplementary Figure S9. Effects of IDRs deletion on the cellular mobility of B23.1. The mobility of EF-B23.1 (aqua), EF-B23.3 (blue), and EF-B23.1-ΔA1A2 (green) proteins expressing in HeLa cells were examined with FRAP assay (left panel). The data of EF-B23.1 and EF-B23.3 were same with Figure 1C, and the data of EF-B23.1-ΔA1A2 was represented as mean values ± SD from 13 experiments.

Supplementary Figure S10. The hypothetical models for inter-molecular interaction between acidic and basic IDRs in full length B23.1. Full length B23.1 forms a pentamer (top left) and decamer (top right) through its N-terminal oligomerization domain. The association of acidic and basic IDRs of B23.1 occur in two molecules in a pentamer (top left) or two pentamers (top right). The illustrations of each region are indicated at the bottom.

Supplementary Figure S1

A

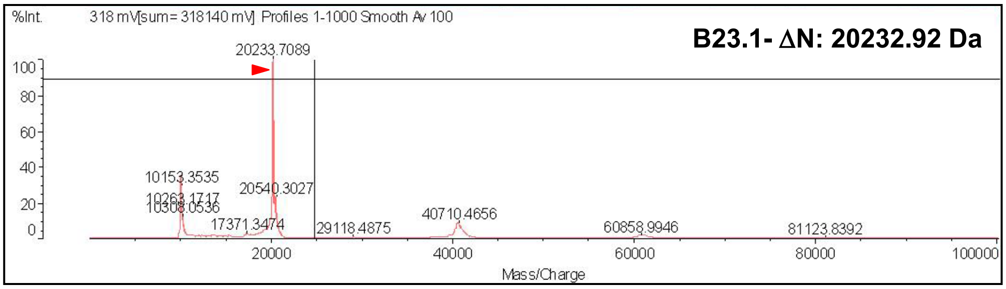

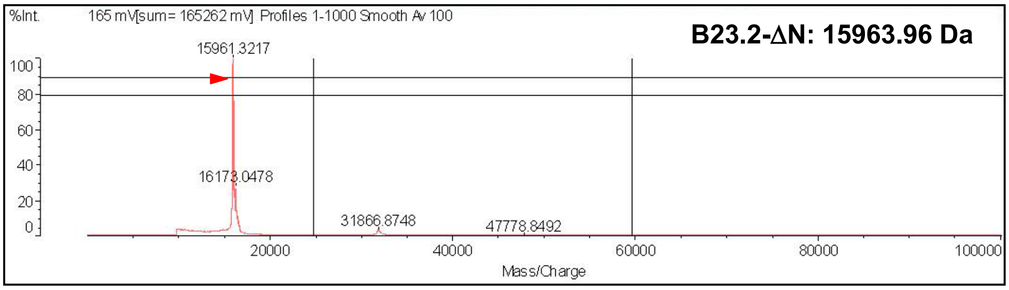

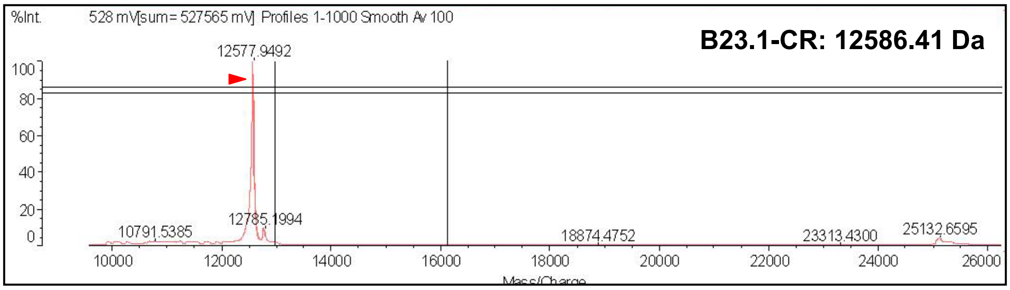

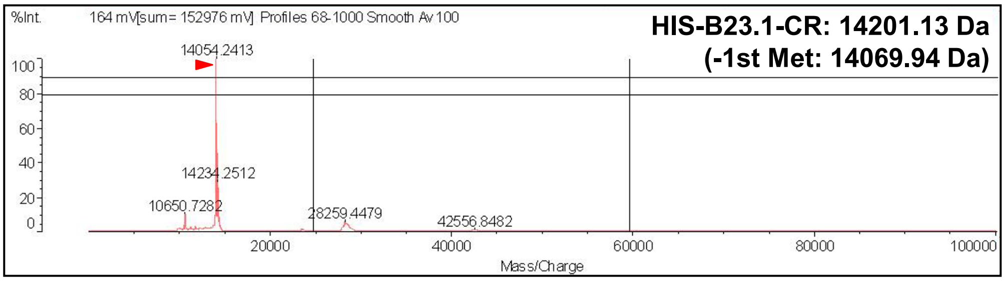

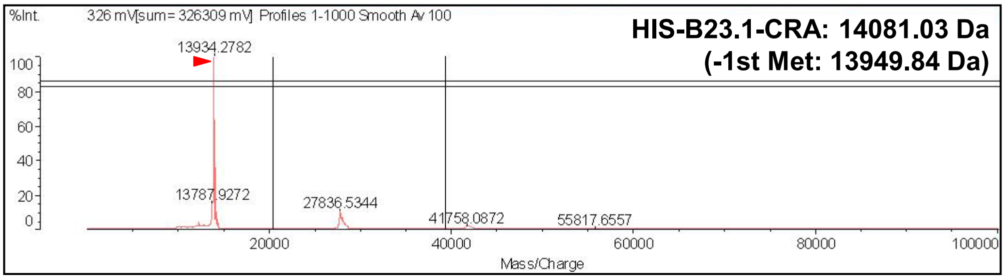

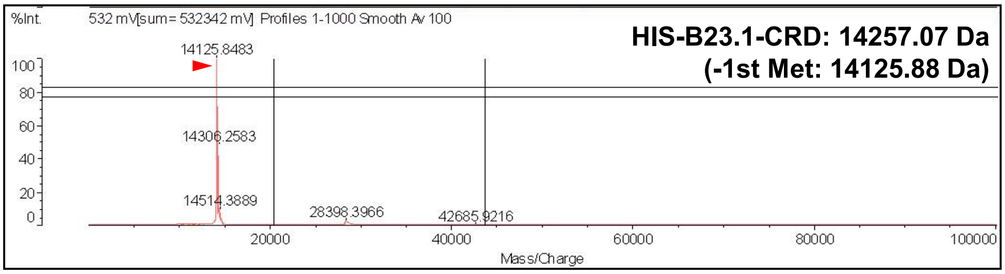

Supplementary Figure S1

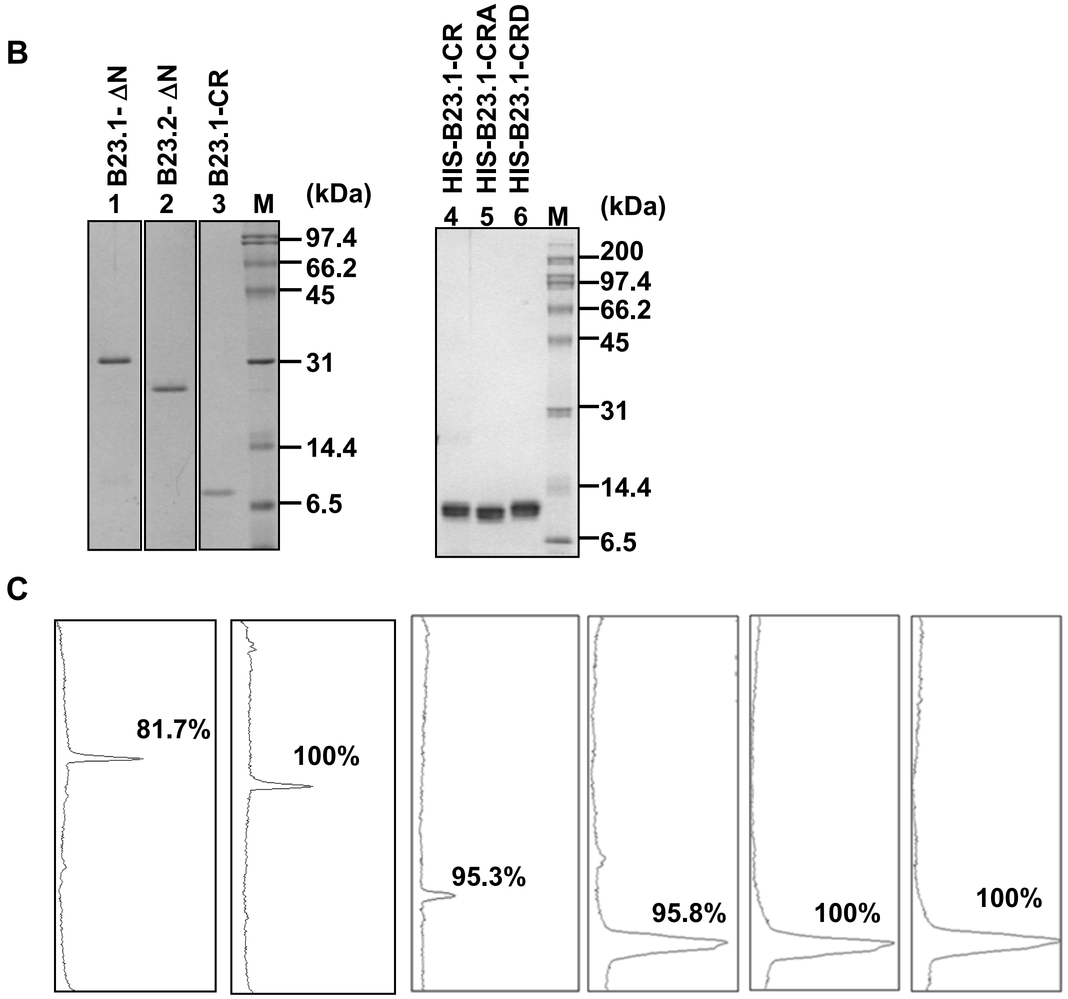

Supplementary Figure S2

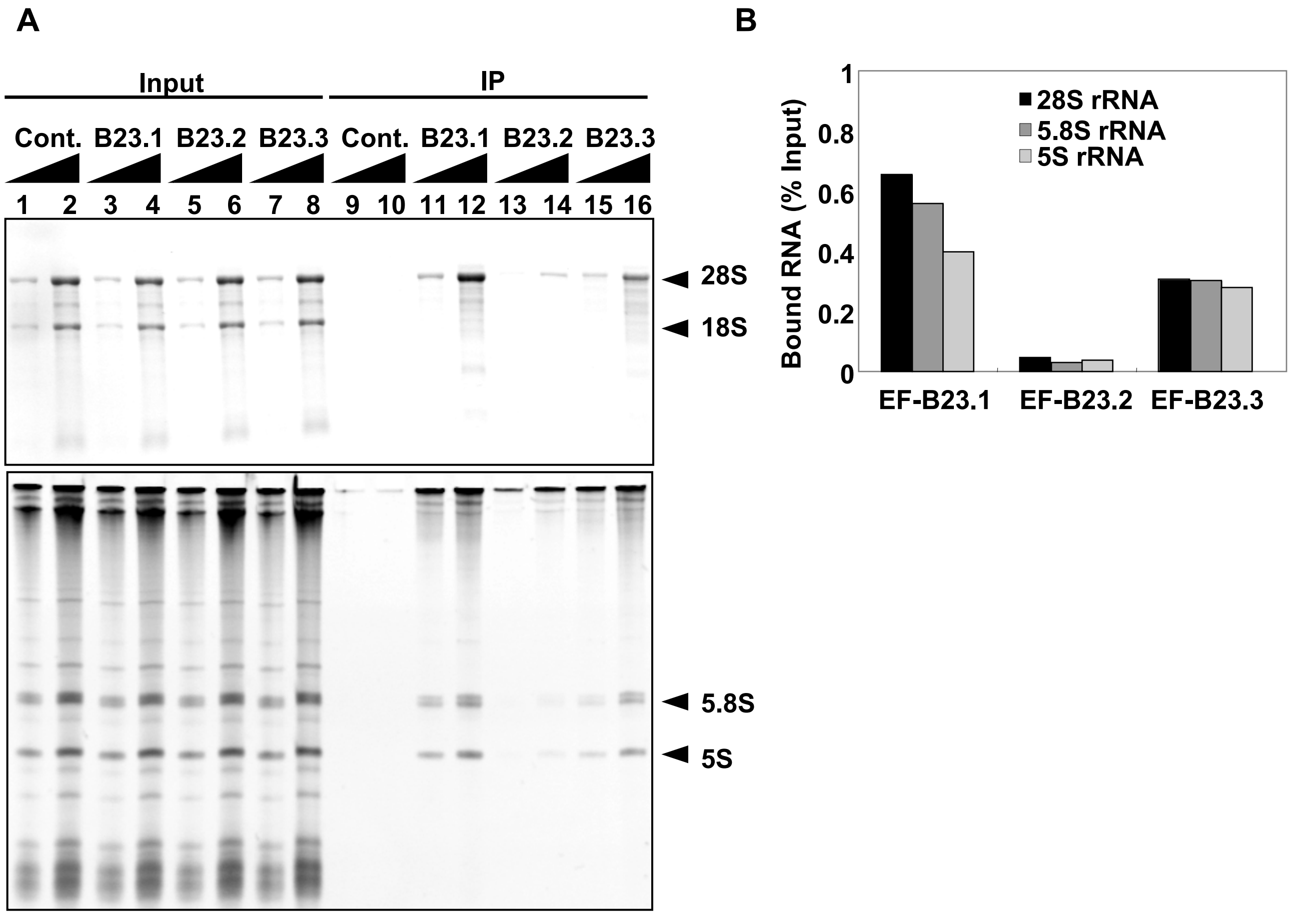

Supplementary Figure S3

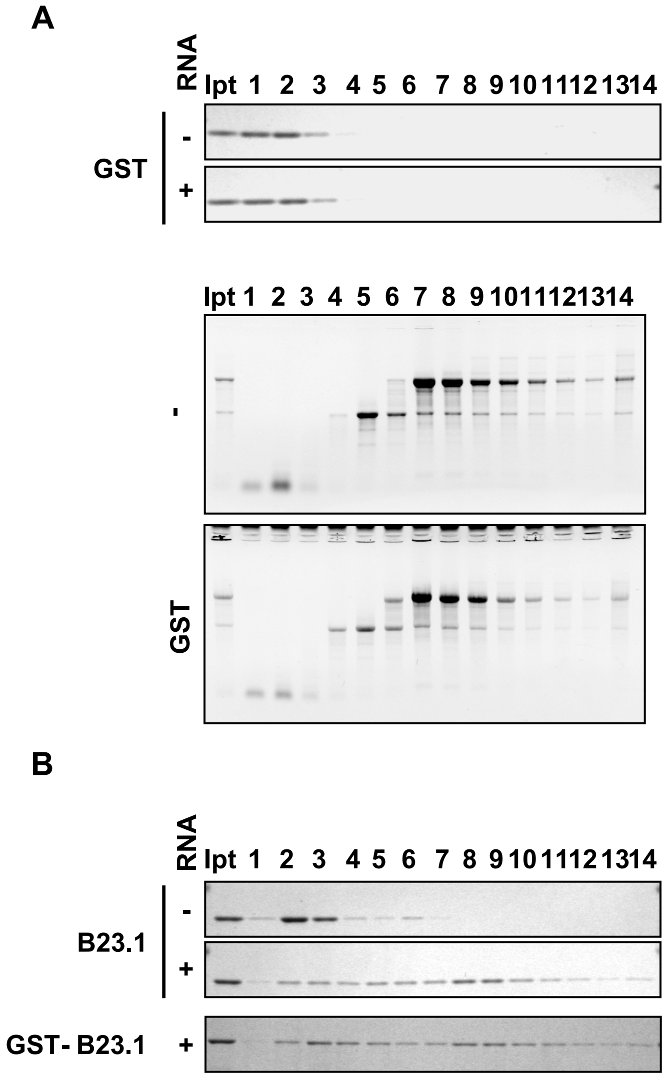

Supplementary Figure S4

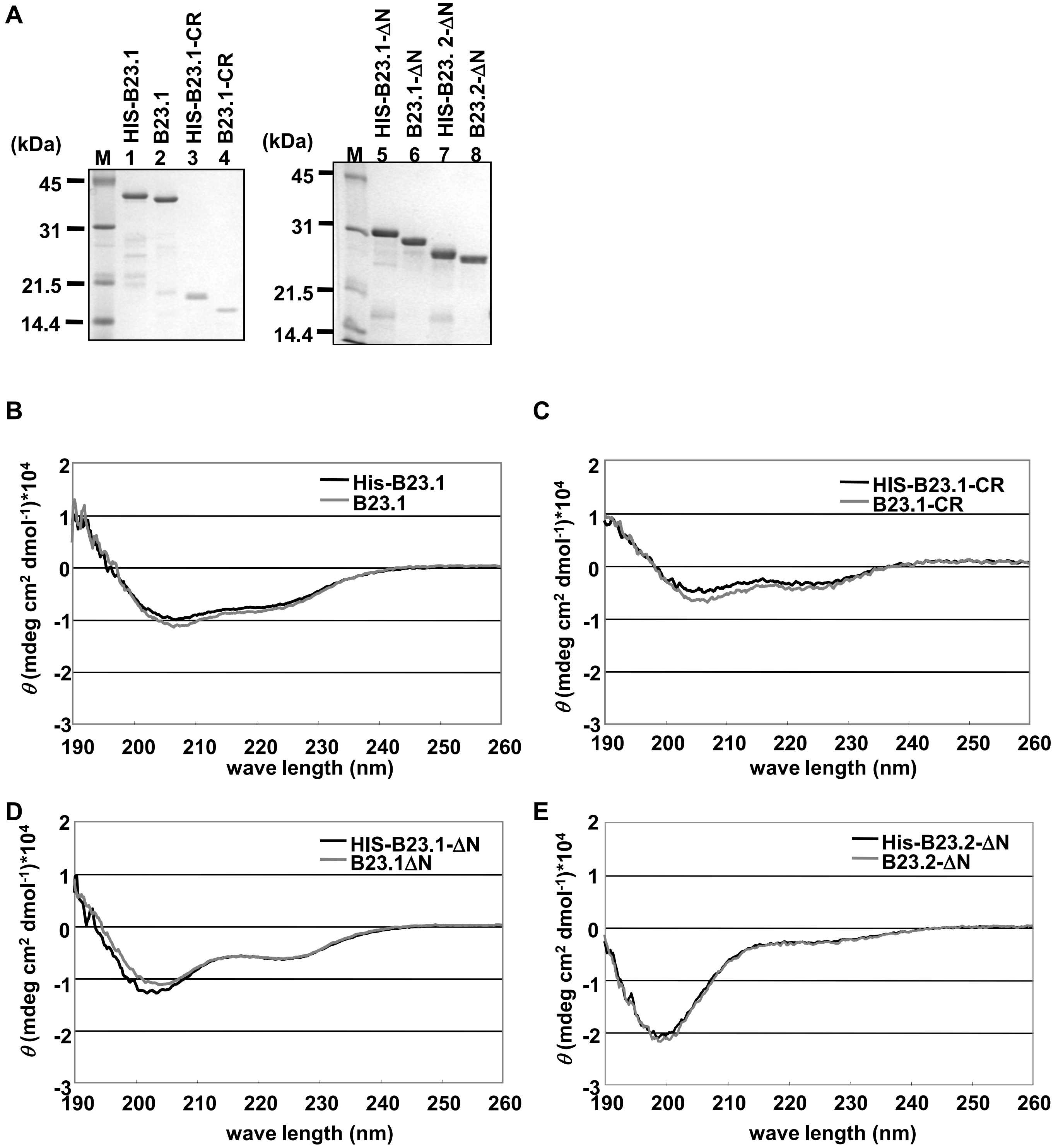

Supplementary Figure S5

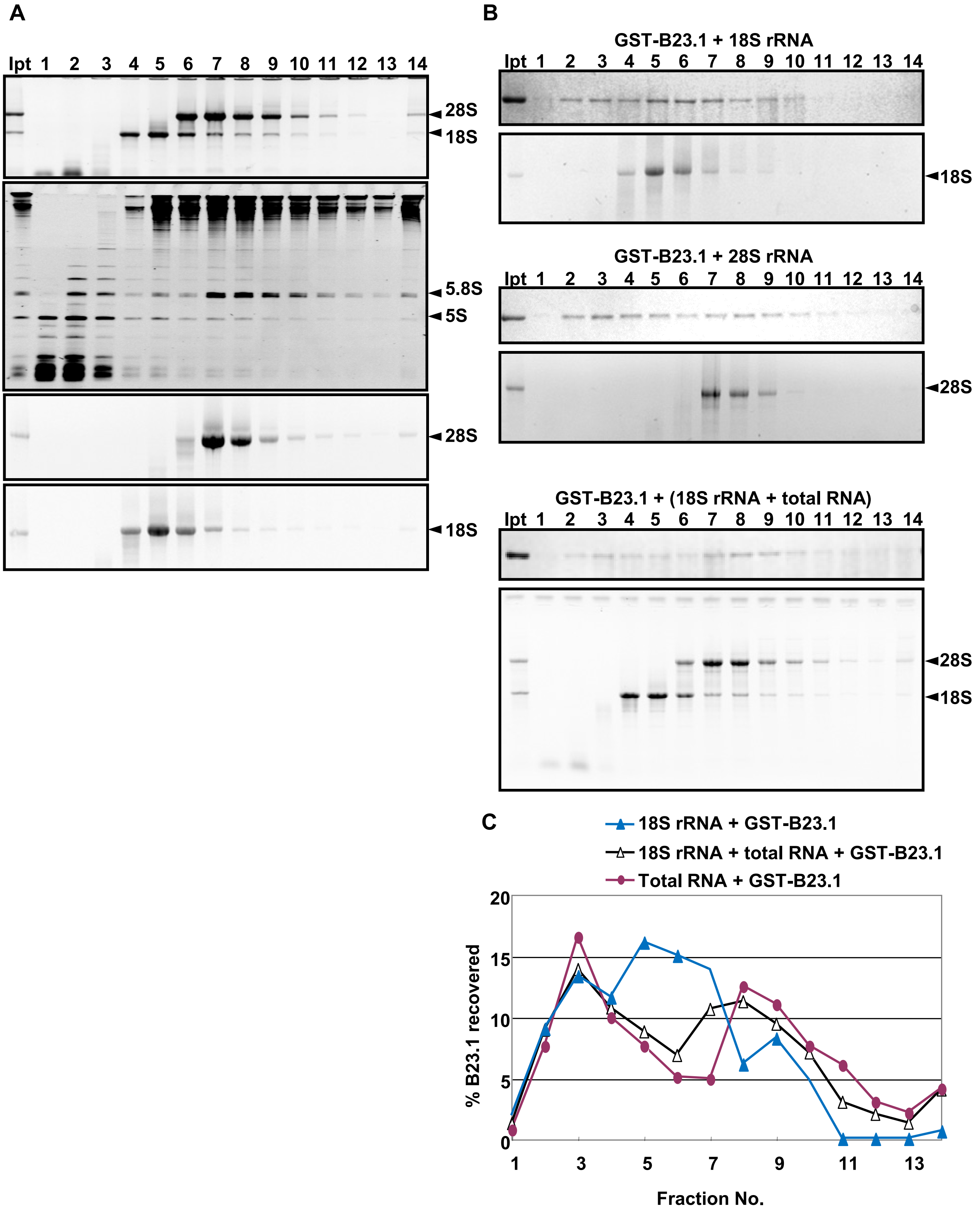

Supplementary Figure S6

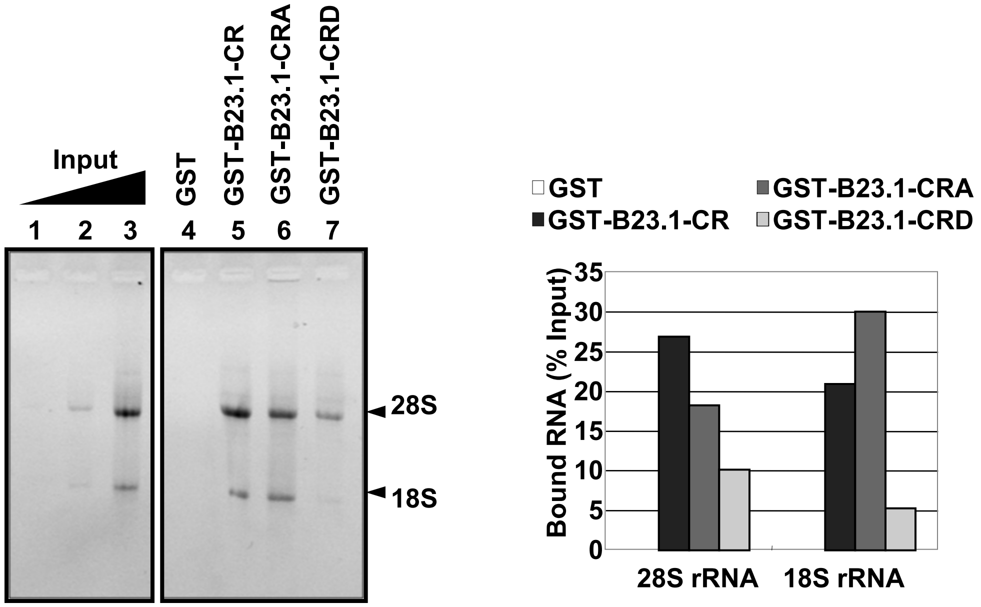

Supplementary Figure S7

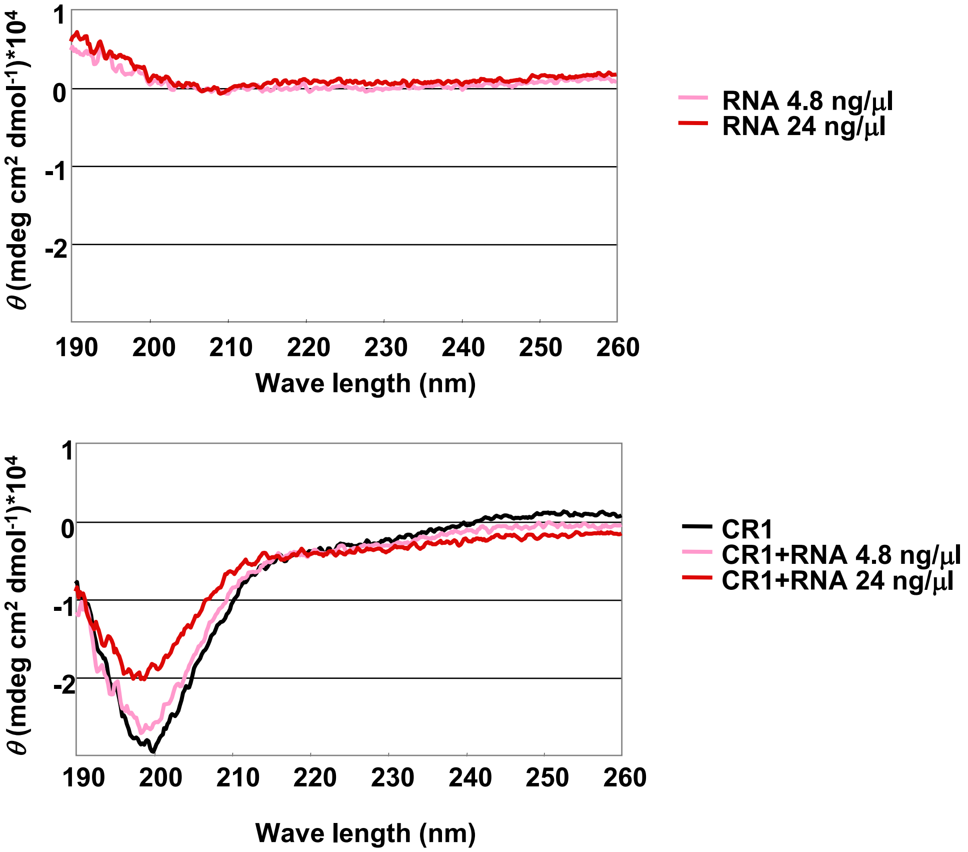

Supplementary Figure S8

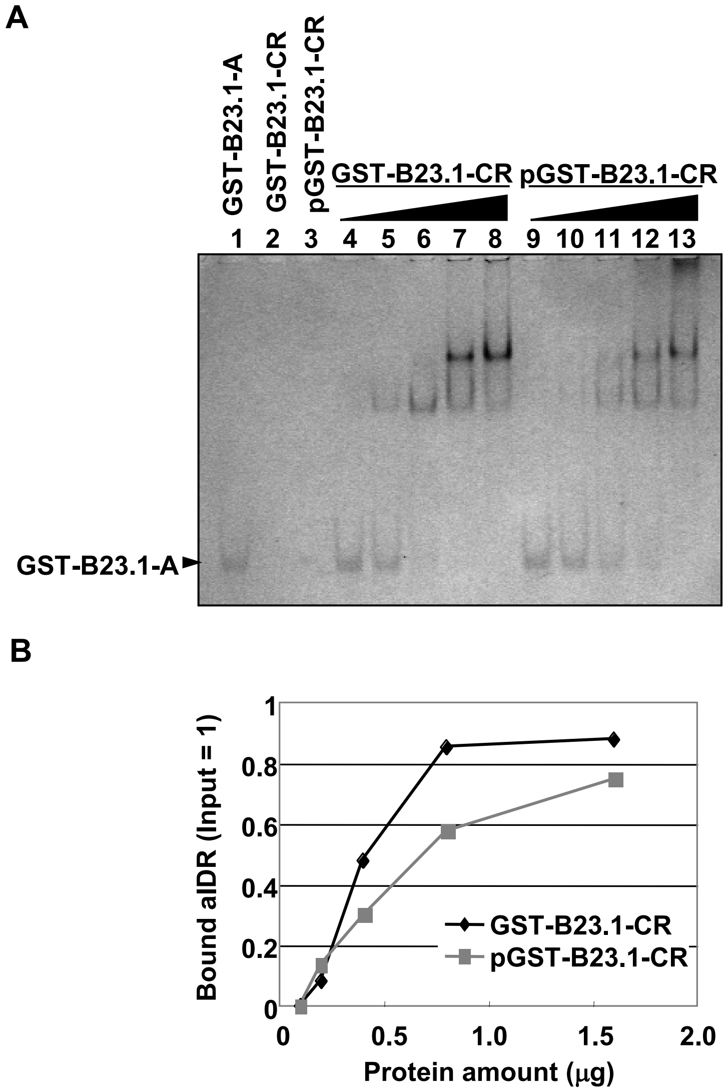

Supplementary Figure S9

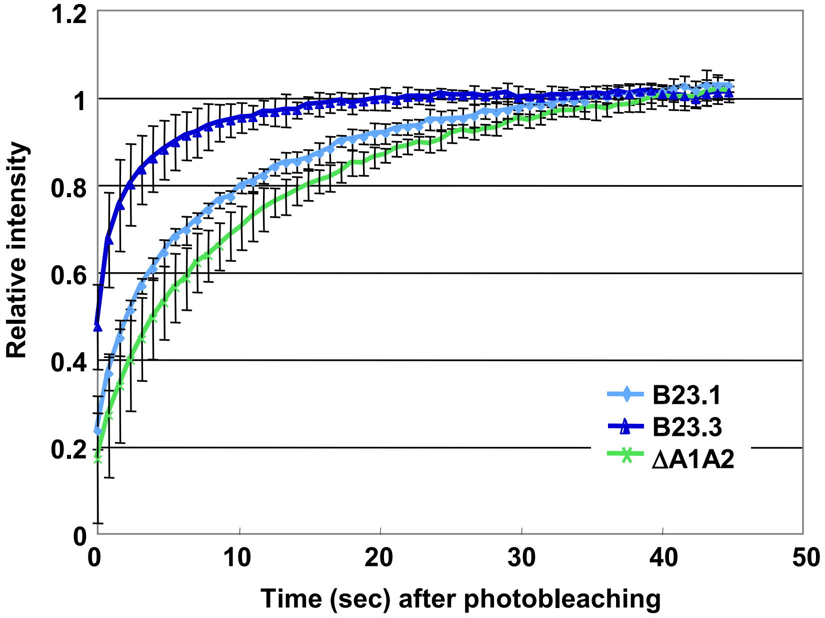

Supplementary Figure S10

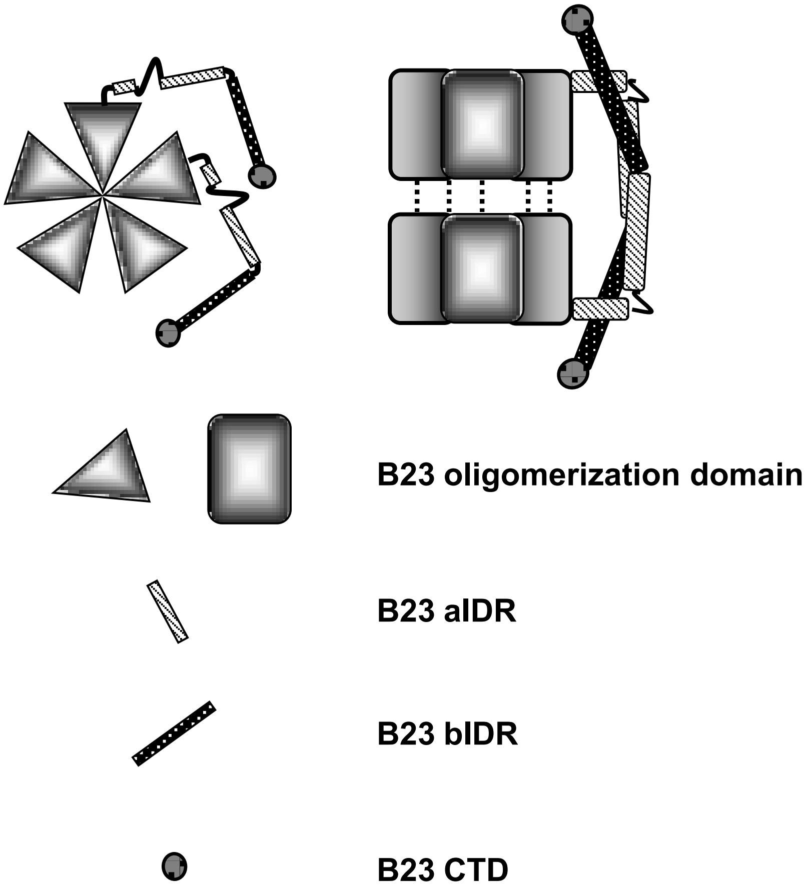

Supplement: Supplementary Data [file supp_gkt897_nar-01624-v-2013-File003.pdf]
